# Does context recollection depend on the base-rate of contextual features?

**DOI:** 10.1007/s10339-023-01153-1

**Published:** 2023-09-11

**Authors:** Marek Nieznański, Michał Obidziński, Daria Ford

**Affiliations:** Institute of Psychology, Cardinal Stefan Wyszyński University, ul. Wóycickiego 1/3 Bud. 14, 01-938 Warsaw, Poland

**Keywords:** Context memory, Base-rate neglect, Conjoint recognition paradigm, Dual-recollection theory, Deep distortions

## Abstract

Episodic recollection is defined by the re-experiencing of contextual and target details of a past event. The base-rate dependency hypothesis assumes that the retrieval of one contextual feature from an integrated episodic trace cues the retrieval of another associated feature, and that the more often a particular configuration of features occurs, the more effective this mutual cueing will be. Alternatively, the conditional probability of one feature given another feature may be neglected in memory for contextual features since they are not directly bound to one another. Three conjoint recognition experiments investigated whether memory for context is sensitive to the base-rates of features. Participants studied frequent versus infrequent configurations of features and, during the test, they were asked to recognise one of these features with (vs. without) another feature reinstated. The results showed that the context recollection parameter, representing the re-experience of contextual features in the dual-recollection model, was higher for frequent than infrequent feature configurations only when the binding of feature information was made easier and the differences in the base-rates were extreme, otherwise no difference was found. Similarly, base-rates of features influenced response guessing only in the condition with salient differences in base-rates. The Bayes factor analyses showed that the evidence from two of our experiments favoured the base-rate neglect hypothesis over the base-rate dependency hypothesis; the opposite result was obtained in the third experiment, but only when high base-rate disproportion and facilitated feature binding conditions were used.

## Introduction

Dual-process models of memory postulate that recognition memory performance reflects the contribution of two distinct components referred to as recollection and familiarity (Yonelinas 2002). Recollection reflects the conscious reinstatement of details from a learning episode, including both target and contextual information, whereas familiarity reflects a more automatic and general activation of a memory trace. A variation of the dual-process view of memory is the fuzzy-trace theory (e.g., Brainerd and Reyna 1990, 2002, 2004), which assumes that two qualitatively different types of representations, verbatim trace and gist trace, are encoded in parallel during a study experience. Verbatim trace stores perceptual item-specific information about a stimulus, whereas gist trace represents more general meaning-based information. Overall, recollection reflects verbatim trace retrieval, whereas familiarity is based on gist trace processing (e.g., Reyna 2012; cf. Nieznański et al. 2019).

Recently, Brainerd and colleagues (Brainerd et al. 2014a; Brainerd et al. 2015) have impugned the unitary view of recollection and proposed a model that distinguishes between the conscious recollection of contextual information and the vivid reinstatement of target information. In this model, target recollection derives from the retrieval of verbatim traces of old items, whereas context recollection is based*—*like familiarity—on gist trace processing. Most recently, however, Brainerd et al. (2022a) have acknowledged that contextual details may be stored in a type of memory trace that is separate from verbatim and gist, namely, a contextual trace. They argued that contextual details are typically associated with multiple old items, which makes them distinct from surface and semantic details specific to particular items. This three-dimensional structure was supported by a meta-analysis of conjoint recognition studies, which distinguished a semantic familiarity (gist trace-based) factor, a context recollection (contextual trace-based) factor, and a target recollection (verbatim trace-based) factor.

Our research stems from an assumption that the strength of the contextual trace can reflect the frequency of occurrence of a particular contextual feature among multiple old items. The more items share the same contextual feature, the stronger the contextual trace of this feature should be. We also hypothesize that the probability that a probe containing a particular contextual feature will evoke context recollection of another associated contextual feature is affected by the frequency of these two contextual features co-occurring. For example, context recollection that a cue word printed in a large font size was green should be higher when most of the presented large-font-size words were printed in green. In other words, we predict that context recollection is sensitive to the base-rate of contextual information experienced during study and reflects the frequency of context-context pairings. This *base-rate dependency account* finds some support in studies on multidimensional source recognition (e.g., Meiser and Bröder 2002) or in studies on ‘pattern completion’ (e.g., Horner et al. 2015; Horner and Burgess 2013). However, there are also some compelling arguments in favour of an alternative view—the *base-rate neglect account*, which refers to the phenomenon known from the judgment and decision-making literature that people have a strong tendency to favour diagnostic information over the base-rates when judging the probability of an event (e.g., Kahneman and Tversky 1973). The aim of the current study is to estimate the evidence in favour of the base-rate dependency hypothesis versus the base-rate neglect hypothesis in the recollection of correlated contextual features.

### Arguments in favour of base-rate dependency in memory

The dependency of context memory on the experienced base-rate of contextual features is consistent with the mutual cuing hypothesis (e.g., Arnold et al. 2019; Boywitt and Meiser 2012; Meiser 2014; Meiser and Bröder 2002) which claims that the successful retrieval of one contextual feature serves as a cue for the other contextual feature. The positive stochastic dependence among concurrent retrieval processes for multiple contextual features observed by Meiser and colleagues suggests that these features are integrated into coherent episodic trace (but see Starns and Hicks 2005; Vogt and Bröder 2007). Encoding events into integrated traces facilitates the joint retrieval of the configurations of features. Importantly, such a dependence was observed when participants declared that they consciously *recollected* the contextual feature (the state of “remembering”) but not in the state based on familiarity (“knowing”). This supports our prediction that the context recollection process, which is defined in the dual recollection theory as a state of vivid reinstatement of contextual features (Brainerd et al. 2014a; Brainerd et al. 2015), is sensitive to the frequency of context-context configurations.

Since the mutual cuing hypothesis predicts that the successful retrieval of one contextual feature facilitates the retrieval of the other contextual feature, the more we can expect such facilitation to occur when one of these features does not need to be retrieved, but is provided to the subject. In such a case, the cueing of the second feature is not conditional on the successful retrieval of the first, but the provided feature is ready for use as a cue. Therefore, in our experiments, we introduced a manipulation of the reinstatement of one of the features as a condition that should enhance base-rate dependency.

The dependency of the retrieval of one element on the retrieval of another element was also demonstrated for elements that are not subordinates, that is, are not contextual features. In the Horner and Burgess (2013) experiments, participants were required to learn *location*-*person*-*object* triplets. The authors analysed how dependent the retrieval of one element (e.g., the person) is on the retrieval of another element (e.g., the object) when cued by a third element (e.g., the location), and they confirmed an interdependence in the ability to retrieve the different elements comprising the same event. Other studies also found support for the view that event elements are integrated into coherent ‘event engrams’ that enable episodic recollection (Horner et al. 2015). Incidental aspects of an event, as contextual details, are also retrieved along with other elements of a complete event. The retrieval of all these constituents of an event when presented with a partial cue is named ‘pattern completion’. The holistic recollection of event elements resulting from their associative structure is even regarded as the defining characteristic of episodic memory, and it was observed both for simultaneously and separately encoded event elements (Horner et al. 2015; James et al. 2020; but see Trinkler et al. 2006).

Research on mutual cuing hypothesis and pattern completion converge in their theoretical conclusions, but use quite different research paradigms, taking this into account, in our Experiment 1 we used a procedure more like that of source memory research (e.g., Meiser and Bröder 2002), while in Experiments 2 and 3 we also used a procedure like that of pattern completion research with colour-object-location triplets (e.g., Horner and Burgess 2013).

Important support for the base-rate dependency in memory also comes from Anderson and Schooler’s (1991) environmental explanation of such memory phenomena as practice, retention, and spacing effects. They describe the memory system as making statistical inferences and reflecting the structure that exists in the environment. According to their observations, the memory system tries to make available those memories that are most likely to be useful in a given time and environment. Therefore, we can expect that memory will also mirror the frequencies of features configurations experienced during the study phase of a memory experiment. This should happen whether or not subjects consciously notice the frequency structure of features, just as awareness of the fact that an event is repeated is not needed for the practice effect to occur.

### Arguments in favour of base-rate neglect in memory

As Johnson et al. (1993) stated in their source monitoring framework, source attributions can be influenced by prior knowledge, schemas, or expectations. The strength of prior associations between features, especially when attentional resources are restricted, may influence item-context binding processes (Nieznański 2013). However, as demonstrated by Bayen et al. (2000), schema-based expectancy seems to influence guessing rather than the ability to remember the source. Source guessing is informed by (a) schema-based bias, which is cross-situational and based on general world knowledge, and (b) probability matching, which is based on situation-specific item-source contingency (e.g., Bell et al. 2020; Spaniol and Bayen 2002). The latter mechanism reflects base-rates experienced at encoding, so that, when source memory is not available, participants guess the source of detected-old items consistently with the proportion of sources associated with the particular type of items (e.g., Bayen and Kuhlmann 2011; Kuhlmann et al. 2012; Wulff et al. 2021). This line of research clearly indicates that specific contingencies of item types and sources influence guessing but not source detection, and this assertion is based on analyses using the two-high threshold multinomial model for source monitoring (Bayen et al. 1996), which enables the separation of the processes of item detection, source discrimination, and response bias. Therefore, the probability-matching account suggests that base-rate dependency appears in metamemory judgments rather than in object-level memory processes.

Base-rate neglect is well-known as one of the many errors and fallacies of human probability judgment, which were initially described in the Kahneman and Tversky research program (e.g., Kahneman and Tversky 1973; Tversky and Kahneman 1983; Tversky and Kohler 1994). In the domain of memory, some analogues of such fallacies were investigated by Brainerd and colleagues. For example, Brainerd et al. (2014b) described conjunction illusions, that is, instances in which participants falsely remember that a target from a single source was presented in multiple sources (see also: Brainerd et al. 2017; Nakamura and Brainerd 2017). In recent reviews, instances when the structure of real-world events is not preserved by our memories were referred to by Brainerd (2021, 2022) as ‘deep distortions’. The study of these phenomena has been inspired by the fuzzy-trace theory’s idea of gist memory, which implies that the retrieval of gist traces supports the acceptance of items belonging to different reality states that are mutually incompatible, for example, a related distractor may be accepted when asked if it is a related new item, but also when asked if it is a target because the target and the related distractor share a gist. Deep distortions are a new family of false memories that operate at a higher level of measurement than surface distortions. Compared to traditional false memories, they are theoretically more fundamental and measurable by analysing relations between two or more memories. Emergent relations among these memories of events or sources, usually studied using the conjoint recognition paradigm, are confronted with certain normative principles and are classified as deep distortions when they violate the axioms and rules of logic or classical probability theory (Brainerd 2021, 2022). An interesting recent example of a violation of the laws of logic comes from the Brainerd et al. (2022b) experiments, which showed that old? and new? judgments do not produce equivalent recognition accuracy. Despite logical equivalence, accuracy levels differ for judgments that an item is old from judgments that it is not new, and judgments that an item is new differ from judgments that it is not old.

Our aim was to analyse relations between memories for frequent versus infrequent configurations of features. It is possible that base-rate neglect in context memory is another example of when the structure of an everyday experience is not preserved by our memories—in this case, our memory does not act on the logic of conditional probability. An attempt to demonstrate the base-rate neglect in source memory was made by Lu and Nieznański (2020), however, that study did not apply modelling analyses to separate the contribution of context recollection.

## Experiment 1: Context memory for equally versus unequally distributed features in neutral and reinstated test conditions

The general goal of Experiment 1 was to ascertain the presence or absence of an effect of an apparent correlation between contextual features on memory for one of these features. All the presented items differed in two dimensions of colour and size. The memory for the colour dimension was tested, and the distribution of colours by font size was manipulated within-subjects. For small-size items, the colours were equally distributed, whereas for large-size items saliently more items were presented in one colour than another. The main question was whether the base-rates experienced during the study influence context memory or do they just affect the guessing bias. For evenly distributed features, the influence of the base-rate should result in the absence of differences in context memory performance, while for disproportionately distributed features, the impact of the base-rate should result in differences in context memory performance. Moreover, if context-to-context associations are encoded into an integrated memory trace, reinstating the item size should reactivate colour memory, resulting in better context memory test performance (e.g., Symeonidou and Kuhlmann 2021, but see Hicks and Starns 2016).

In the condition with the reinstated large or small font size at the test in comparison with the condition with the neutral (medium) font size, applying the (implicit or explicit) knowledge about the correlation between contextual features should be easier. In this condition, participants were directly informed that the font used to present the word at the test is the same size as the font used at the study, therefore, they can use their knowledge about the base-rates of colours in particular fonts (e.g., that words printed in green were often presented in large font and rarely in small font). Applying the learnt correlation between features is also possible in the neutral condition depending on the ability to spontaneously mentally reinstate the font size of the presented word (cf. Starns and Hicks 2013). However, since the study font size may be forgotten or falsely attributed, context memory in the neutral condition should be much less affected by the base-rates than in the condition with the reinstated font.

### Participants

In this experiment, 78 participants were recruited from among first and second-year psychology undergraduates. They received extra credits in their courses. One participant was excluded since he reported colour blindness. Participants’ mean age was 20.93 years (*SD* = 3.46), 18 were men.

### Stimuli

As the materials, we used 123 nouns in Polish taken from the dataset prepared by Imbir (2016). According to the ratings available in this dataset, the selected words were all low in arousal, of medium valence and frequency, and of medium or high imaginability. In detail, our materials met the following criteria: all were nouns, 4–6 letters long, with a valence rating (on a scale from 1 to 9) between 3 and 7, an arousal rating lower or equal to 3.6, imaginability higher or equal to 4; and a frequency of appearance in the language from 300 to 1500 (Mandera et al. 2014).

### Procedure and design

The participants were examined at individual workstations in the University Lab. The presentation of the stimuli and the response recording were controlled using the E-Prime 2.0 program (Psychology Software Tools, Pittsburgh, PA).

**Fig. 1:**
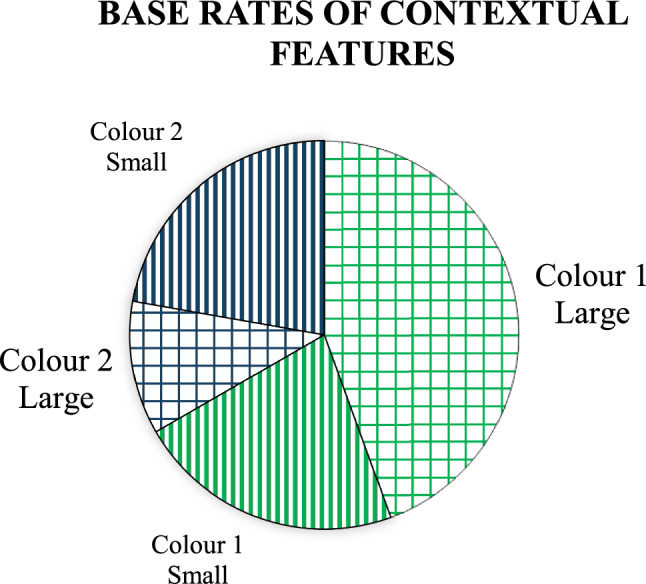
A circle diagram illustrating the proportions of contextual features presented at the study phase of Experiment 1. Green areas depict words in Colour 1, blue areas in Colour 2, lattices represent words in large font, and vertical lines represent words in small font

At study, 81 words were presented, two-thirds of them (54) were presented in font Colour 1, and one-third (27) in font Colour 2, thus, the base-rates were manipulated within subjects. For approximately half of the participants, Colour 1 was green and Colour 2 was blue, and vice versa for the other half. The participants were asked to try to remember words along with their colour and size. They were notified that some colours are more frequent in a particular font size than in another. The words were presented in a random order, at a rate of 4 s, with an interstimulus interval of 250 ms. Among 81 words, 45 were presented in large font size (96 pts) and 36 in small font size (24 pts); the font type was *Arial*, bold. Among 54 words in Colour 1, two-thirds (36) were presented in large and one-third (18) in small font size. Among the 27 words in Colour 2, one-third (9) were presented in large and two-thirds (18) in small font size. Overall, there were more Colour 1 than Colour 2 words, and Colour 1 words were more often in large font than small font, and the opposite was true for Colour 2 words. Figure 1 illustrates the proportions of words in each colour and font. Formally, the probability of a particular colour given a particular font size can be computed using the conditional probability formula, as follows:

$$P\left({C}_{1}|L\right)=\frac{P(L\cap {C}_{1})}{P(L)}= \frac{36/81}{45/81}=0.8,$$

$$P\left({C}_{1}|S\right)=\frac{P(S\cap {C}_{1})}{P(S)}= \frac{18/81}{36/81}=0.5,$$

$$P\left({C}_{2}|L\right)=\frac{P(L\cap {C}_{2})}{P(L)}= \frac{9/81}{45/81}=0.2,$$

$$P\left({C}_{2}|S\right)=\frac{P(S\cap {C}_{2})}{P(S)}= \frac{18/81}{36/81}=0.5.$$

where C_1_ = Colour 1, C_2_ = Colour 2, L = large font, and S = small font. Therefore, when a particular test probe is recognized as being presented at study in the large font, it is also expected that it was presented in Colour 1 rather than Colour 2 (the a priori hypothesis that it was Colour 1 is 4 times more probable than that it was Colour 2). However, when a word is presented in small font at test, it is equally probable that it was in Colour 1 or 2 at study.

At test, the studied words were presented intermixed with 42 distractors. Reinstatement of font size at test was manipulated between subjects. The words were presented in the same—large (96 pts) or small (24 pts)—font size for 37 participants, and in a new medium (48 pts) font size for 40 participants. In the reinstated condition, half of the distractors were presented in large font and the other half in small font. At test, the participants were informed that their task was to recognize if the word was presented and answer “yes” or “no” to the question that will be shown under the test word on a particular slide. There were three types of probe questions counterbalanced across participants and presented equally often with each type of test items: (a) Was this word presented in Colour 1?; (b) Was this word presented in Colour 2?; and (c) Was this word presented in either Colour 1 or 2? The slides were presented in random order. The test trials were participant-paced with the next trial appearing immediately after a response.

### Data analysis

Bayesian analyses were conducted in JASP (JASP Team 2019; jasp-stats.org, see: van Doorn et al. 2020). We used Bayes factor BF_10_ to compare the predictive performance of an alternative hypothesis over a null hypothesis. A Bayes factor between 1 and 3 is considered weak evidence, between 3 and 10 moderate evidence, and above 10 is considered strong evidence in favour of an alternative hypothesis. In symmetry, a BF_10_ lower than 1 supports a null hypothesis, a factor between 0.333 and 0.1 means moderate evidence, and below 0.1 is considered strong evidence for a null hypothesis. When the dependent variables were normally distributed and the variances were homogenous across the groups, we performed Bayesian *t* tests, otherwise, we reported the Mann–Whitney *U*-test or the Wilcoxon rank-signed test. As priors we used default options in JASP, that is, the Cauchy distribution with *r* set to $1/\sqrt{2}$.

Multinomial modelling analyses were based on hierarchical Bayesian modelling using the latent-trait approach (Klauer 2010). This approach uses the multivariate normal distribution of the transformed individual parameters as the prior distribution on a group level. Monte Carlo Markov Chain sampling methods are employed to obtain the parameter posterior estimates (for more information about hierarchical multinomial processing tree models and examples of their application see: Arnold et al. 2019; Ernst et al. 2019; Heck et al. 2018; Klauer 2010). All hierarchical multinomial modelling analyses were conducted using the R package TreeBUGS (Heck et al. 2018).

### Multinomial model for conjoint recognition paradigm

In the present research, the multinomial dual-recollection model (Brainerd et al. 2015) was used as a measurement model. The original model was developed for a context memory experiment with targets presented on List 1 and List 2 at study, and with three types of probe questions presented during the test phase: “Was it on List 1?”, “Was it on List 2?” or “Was it on either List 1 or List 2?”. The model defines the following retrieval processes: (a) The *RT*_*1*_ (*RT*_*2*_) parameter (target recollection), which is the probability that a List 1 (List 2) target cue provokes the conscious reinstatement of its presentation during the study; (b) The *RC*_*1*_ (*RC*_*2*_) parameter (context recollection), which is the probability that a List 1 (List 2) target cue provokes the conscious reinstatement of the contextual details of List 1 (List 2) presentation; and (c) The *F*_*1*_ (*F*_*2*_) parameter (familiarity), which represents the probability that a List 1 (List 2) target cue provokes a sufficiently high familiarity to make the target be perceived as old. Moreover, two response bias parameters are also defined: one (*b*) for accepting non-retrieved items (targets or distractors) for List 1? probe questions or List 2? probe questions, and another (*b*_*12*_) for accepting non-retrieved items for List 1 or 2? probe questions (see Brainerd et al. 2015, Table 2). In comparison with the original model, the present research replaced the List 1 targets and the List 2 targets with the targets presented in Colour 1 or Colour 2.

**Fig. 2:**
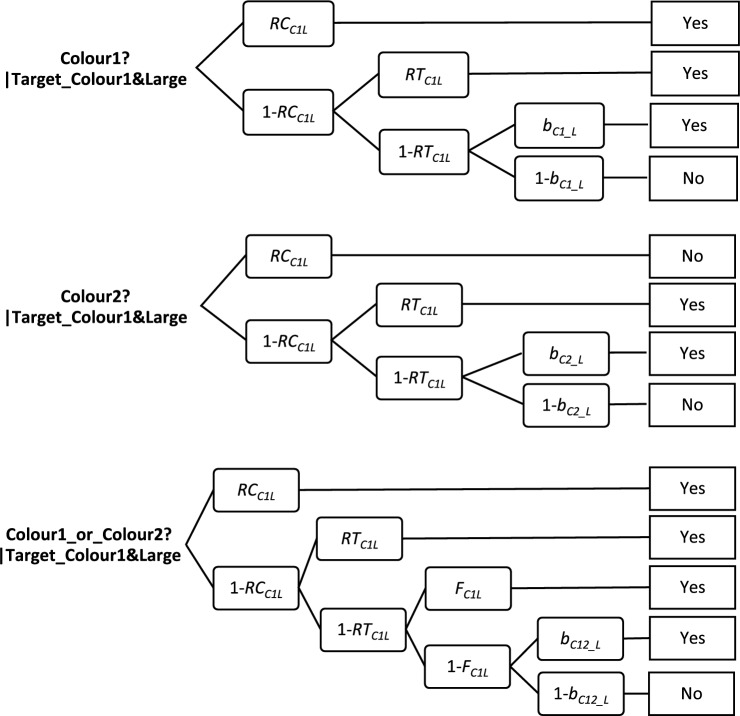
A part of the multinomial dual-recollection model used in Experiment 1 (based on Brainerd et al. 2015, Fig. 1). Colour1?, Colour2?, and Colour1 or Colour2? refer to probe questions. *RC* is a context recollection parameter, *RT* is a target recollection parameter, *F* is a familiarity parameter, and *b* is a response bias parameter. Subscripts indicate the kind of target determined by the font size and colour

A part of the multinomial model applied in the current research is presented in Fig. 2. One tree can be depicted for each probe question and item type. In Fig. 2, only the model of processing of targets presented in Colour 1 and large font is shown as an example. On the left are the item types used at the test with the specified question probes (Colour 1?, Colour 2?, and Colour 1 or Colour 2?). On the right are the participants’ responses (accept or reject), which are connected with the question probes and the item types by the branches of the processing trees representing the latent cognitive processes postulated by the dual recollection theory. As can be seen in Fig. 2, when a target context is congruent with the question probe (C1?|Target_C1), the target cues are accepted if the context recollection (*RC*_*1*_) or the target recollection (*RT*_*1*_) is successful and, if neither are successful, the response bias (*b*_*1*_) can produce a “yes” response. When a target context is incongruent with the question probe (C2?|Target_C1), the target cues are rejected if the context recollection is successful but are accepted if the context recollection fails (1 − *RC*_*1*_) and the target recollection (*RT*_*1*_) is successful, and a “yes” response may also be produced by the response bias (*b*_*2*_). On the probes with the Colour 1 or Colour 2? question (C1or2?|Target_C1), the participants respond “yes” if the context recollection, target recollection or familiarity (*F*_*1*_) are successful; and if all of these retrieval processes fail, the response bias (*b*_*12*_) can produce acceptance. For distractors, only the response bias (*b*_*1*_ for C1?, *b*_*2*_ for C2?, and *b*_*12*_ for C1or2?) can produce acceptance (cf. Brainerd et al. 2015). Separate models of this type were created for large and small font-size items.

## Results

### Results based on descriptive measures

**Fig. 3:**
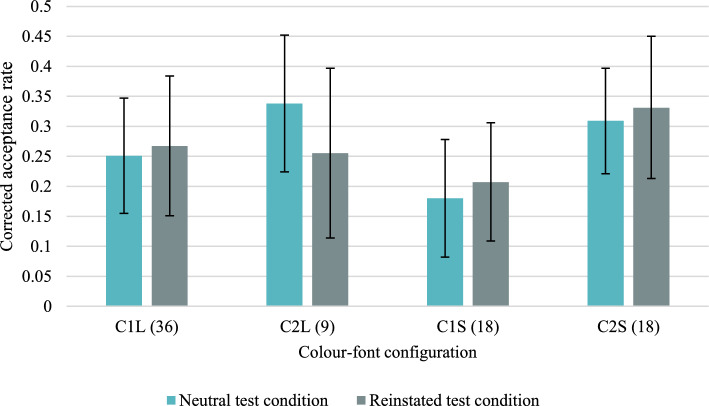
Mean corrected-for-guessing acceptance rates for accurate colour recognition for configurations of contextual features in Experiment 1. Error bars represent 95% credible intervals. Numbers in parentheses indicate the number of items in the condition

**Fig. 4:**
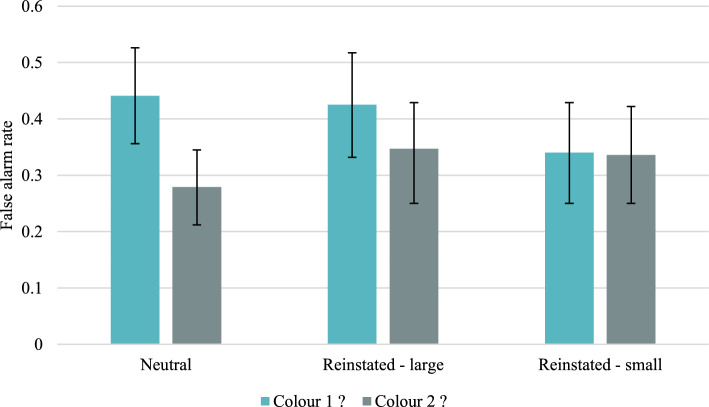
Mean false alarm rates in Experiment 1. Error bars represent 95% credible intervals

Descriptive statistics concerning the mean acceptance rates and the mean corrected acceptance rates (CAR) (i.e., the probability of a “yes” response for targets minus the probability of a “yes” response for distractors) for particular colour/font configurations and for each type of probe question are presented in Tables 14 and 15 in the “Appendix 1”. Figure 3 presents only the means and 95% credible intervals of accurate CARs, and Fig. 4 presents false alarms for distractors. The grand means of accurate CARs, compared between large font size items (*M* = 0.278, *SD* = 0.358) and small font size items (*M* = 0.256, *SD* = 0.313), pooling over configuration types and test conditions, were not significantly different, *t*(153) = 0.82. However, when the grand means of accurate CARs were compared between Colour 1 items (*M* = 0.226, *SD* = 0.312) and Colour 2 items (*M* = 0.309, *SD* = 0.354), a significant difference was found, *t*(153) = 2.81, Cohen’s *d* = 0.23, *p* = 0.006, indicating that participants attributed the less frequently presented colour more accurately.

**T﻿able 1:** Group-level parameter estimates (standard deviations) and 95% Bayesian Credible Intervals of the dual-recollection multinomial model obtained in Experiment 1

	Large font		Small font	
	Colour 1 (n = 36)	Colour 2 (n = 9)	Colour 1 (n = 18)	Colour 2 (n = 18)
Neutral condition				
*RT*	0.304 (0.041) [0.222, 0.383]	0.324 (0.073) [0.169, 0.457]	0.230 (0.047) [0.131, 0.318]	0.277 (0.048) [0.177, 0.366]
*RC*	0.185 (0.054) [0.077, 0.289]	0.219 (0.068) [0.078, 0.346]	0.111 (0.061) [0.012, 0.239]	0.143 (0.063) [0.030, 0.272]
*F*	0.127 (0.077) [0.008, 0.289]	0.352 (0.161) [0.042, 0.647]	0.297 (0.130) [0.039, 0.530]	0.121 (0.082) [0.005, 0.300]
*b*_*C1*_	0.407 (0.045) [0.318, 0.493]			
*b*_*C2*_	0.250 (0.034) [0.185, 0.319]			
*b*_*C1or2*_	0.224 (0.027) [0.171, 0.279]			
Reinstated condition				
*RT*	0.287 (0.056) [0.169, 0.390]	0.295 (0.092) [0.090, 0.457]	0.275 (0.048) [0.174, 0.363]	0.340 (0.083) [0.169, 0.498]
*RC*	0.167 (0.062) [0.051, 0.290]	0.097 (0.068) [0.005, 0.253]	0.046 (0.036) [0.002, 0.133]	0.108 (0.062) [0.011, 0.241]
*F*	0.142 (0.102) [0.007, 0.375]	0.396 (0.183) [0.047, 0.740]	0.186 (0.110) [0.014, 0.418]	0.068 (0.064) [0.001, 0.230]
*b*_*C1*_	0.398 (0.047) [0.304, 0.488]		0.309 (0.052) [0.208, 0.410]	
*b*_*C2*_	0.287 (0.052) [0.187, 0.387]		0.322 (0.049) [0.224, 0.415]	
*b*_*C1or2*_	0.228 (0.048) [0.138, 0.323]		0.260 (0.045) [0.173, 0.351]	

The italicized symbols are the parameters of the dual-recollection multinomial model: *RT* = target recollection, *RC* = context recollection, *F* = familiarity, and *b*_*C*_ = response bias for Colour1?, Colour2? or Color1 or Colour2? probe questions

**Table 2:** Differences in the mean dual-recollection multinomial model parameters and 95% BCIs between the neutral and reinstated conditions

Parameter (neutral–reinstated)	Large font		Small font	
	Colour 1 (n = 36)	Colour 2 (n = 9)	Colour 1 (n = 18)	Colour 2 (n = 18)
Δ*RT*	− 0.008 [− 0.182, 0.138]	− 0.001 [− 0.262, 0.261]	− 0.056 [− 0.200, 0.094]	− 0.117 [− 0.356, 0.078]
Δ*RC*	− 0.042 [− 0.302, 0.152]	0.090 [− 0.171, 0.282]	0.039 [− 0.142, 0.191]	− 0.010 [− 0.279, 0.188]
Δ*F*	− 0.059 [− 0.497, 0.208]	− 0.061 [− 0.562, 0.422]	0.068 [− 0.371, 0.406]	0.020 [− 0.353, 0.249]

Δ*RT* = the difference in the target recollection parameter estimates, Δ*RC* = the difference in the context recollection parameter estimates, Δ*F* = the difference in the familiarity parameter estimates

#### Within-subjects effects of the base-rates

If memory is informed by base-rates, the accurate mean CAR should be higher for the most frequent configuration (Colour 1 and large font size) than the least frequent configuration (Colour 2 and large font size), therefore, the one-sided alternative hypothesis is CAR_C1L_ > CAR_C2L_. However, in the neutral condition, the Bayesian *t* test yielded strong evidence for a null hypothesis, BF_+0_ = 0.078, and, in the reinstated condition, the evidence for a null hypothesis was moderate, BF_+0_ = 0.207. This suggests that memory was not informed by base-rates.

A second comparison was conducted between equally frequent configurations of Colour 1 and Colour 2 with small font size. This time, if memory is informed by base-rates, we should find evidence for the null hypothesis. The alternative two-sided hypothesis is that CAR_C1S_ ≠ CAR_C2S_. In the neutral condition, we found moderate evidence for an alternative hypothesis, BF_10_ = 3.112. In the reinstated condition, the evidence was indeterminate, BF_10_ = 1.096. Therefore, in the case of equally frequent colour/size configurations, the evidence is against the use of base-rates in the neutral condition, and it is inconclusive in the reinstated condition. If the participants ignore the base-rates of the colour/size configurations but they use the general proportion of colours, the alternative hypothesis should be one-sided, CAR_C1S_ > CAR_C2S_, since there were, in general, more items in Colour 1. However, in the neutral condition, we found strong evidence for the null hypothesis, BF_+0_ = 0.052, which means that the data is about 19 times more likely under the null hypothesis.

In the case of false alarms (FA), that is, “yes” responses to distractors (see Fig. 4), if the response bias is informed by base-rates, more “yes” responses should be found for distractors when the participants are asked about the more frequent Colour 1. In the neutral condition, the Wilcoxon signed-rank test yielded Bayes factor, BF_−0_ = 387.375, indicating very strong evidence for the one-sided alternative hypothesis, FA_C2?_ < FA_C1?_. In the reinstated condition, the alternative hypotheses differ depending on the font size in which the distractors were presented. In comparison with the neutral condition, this time the participants need to consider not only which colour was more frequent but also what were the proportions of colours depending on the font size. We found weak evidence for the one-sided alternative hypothesis, FA_LargeC2?_ < FA_LargeC1?_, when the distractors were presented in large font size, BF_−0_ = 1.959. However, when the distractors were presented in small font, for which colours were equally distributed at study, the evidence was moderate for the null hypothesis, FA_SmallC2?_ = FA_SmallC1?_, BF_10_ = 0.186. These results suggest that the response bias is informed by base-rates in both the neutral and reinstated test conditions.

#### Between-subjects effects of context reinstatement

We can hypothesise that the participants in the reinstated condition should be more informed by base-rates than the participants in the neutral condition. Therefore, for the most frequent configuration (Colour 1 and large font size), the mean CAR should be lower in the neutral than in the reinstated condition, CAR_C1Lneutral_ < CAR_C1Lreinstated_. However, we found moderate evidence for the null hypothesis, BF_−0_ = 0.280, from the independent one-sided *t* test. In contrast, the mean CAR should be higher in the neutral than in the reinstated condition for the least frequent configuration (Colour 2 and large font size), CAR_C2Lneutral_ > CAR_C2Lreinstated_. We found weak evidence for the null hypothesis, BF_+0_ = 0.556. Therefore, we cannot conclude that in the reinstated condition the base-rates inform memory more than in the neutral condition, we have moderate or weak evidence for the opposite hypothesis.

### Results based on process measures

Hierarchical analyses were conducted using the latent-trait approach (Klauer 2010) implemented in the TreeBUGS software (Heck et al. 2018). Model fit was assessed with the *T1* (the distance between the observed and the expected mean frequencies) and *T2* (the summed distance between the observed and the expected covariance statistics) (Klauer 2010; see Heck et al. 2018). Good model fit was indicated by nonsignificant test results in the neutral condition (*T*_*1*_: *p* = 0.519, *T*_*2*_: *p* = 0.366) and in the reinstated condition (*T*_*1*_: *p* = 0.446, *T*_*2*_: *p* = 0.424). Group-level dual-recollection multinomial model parameter estimates and their 95% Bayesian Credible Intervals (BCI) are presented in Table 1.

#### Within-subjects effects of base-rates on context recollection and response bias

For each memory parameter, the posterior samples obtained for one configuration of features were subtracted from those obtained for another configuration. Parameters for which the 95% CI of the difference estimates overlapped with 0 do not meaningfully differ between conditions (Smith and Batchelder 2010). In the neutral condition, we compared the context recollection parameter and the response bias depending on the base-rates. The difference in the mean context recollection parameters Δ*RC* between frequent versus infrequent configuration (*RC*_*C1L*_ − *RC*_*C2L*_) was *M* =  − 0.014, with the credibility interval of the difference [− 0.191, 0.189] indicating no substantial effect. Similarly, the difference in the mean parameters between the equally frequent configurations (*RC*_*C1S*_ − *RC*_*C2S*_) indicated no substantial effect, *M* =  − 0.043 with 95% CI [− 0.215, 0.129]. However, in the case of the difference of the response bias Δ*b* depending on the probe question about the frequent versus less frequent colour (*b*_*C1*_ – *b*_*C2*_), a substantial effect was indicated by the credibility interval not overlapping with 0, *M* = 0.149, 95% CI [0.056, 0.238].

In the reinstated condition, the results for the context recollection parameters indicated no substantial effect. In detail, the difference in the mean parameters Δ*RC* were *M* = 0.153, 95% CI [− 0.092, 0.396] for the frequent versus infrequent configuration (*RC*_*C1L*_ − *RC*_*C2L*_), and *M* =  − 0.074, 95% CI [− 0.272, 0.110] for equally frequent configurations (*RC*_*C1S*_ − *RC*_*C2S*_). For the response biases, the 95% CI of the difference estimates overlapped with 0, both when the distractors were presented in large font Δ*b*_*L*_ (*b*_*C1L*_ – *b*_*C2L*_), *M* = 0.097, 95% CI: [− 0.045, 0.215] and when distractors were presented in small font Δ*b*_*S*_ (*b*_*C1S*_ – *b*_*C2S*_), *M* =  − 0.03, 95% CI [− 0.186, 0.117]. This suggests that the response bias did not meaningfully differ between the probes in the reinstated condition.

#### Between-subjects effects of context reinstatement

In this section, we present the results for all the memory parameters of the dual-recollection model since source memory literature suggests that context reinstatement can influence verbatim trace retrieval (Nieznański and Tkaczyk 2017), which has been represented in the model by the target recollection parameter. For each memory parameter, the posterior samples obtained in the reinstated condition were subtracted from those obtained in the neutral condition. As Table 2 shows, no substantial effect of the test condition was indicated for differences in memory parameters.

#### Bayesian analyses of hypotheses about context recollection and response bias

Although hierarchical models specify the parameters both for the group and the individual participants level (Heck, et al. 2018), entering the individual estimates as input into a Bayesian *t* test is problematic.^1^ This is because parameter estimates for individual participants are informed by the group means, especially in the case of less reliable estimates (a property called shrinkage); in result, the estimation error is artificially decreased (Boehm et al. 2018). Therefore, we computed the independent estimates of the parameters for each participant in a conventional way using the maximum likelihood fitting method (e.g., Riefer and Batchelder 1988) implemented in the *multiTree* software (Moshagen 2010). It must be noted, however, that estimates based on relatively few observations per participant do not allow the parameters to be estimated precisely, which increases the error variance. The full results of the maximum likelihood analyses are presented in "Appendix 2".

In the neutral condition, a one-sided Bayesian Wilcoxon signed-rank test yielded moderate evidence for the null hypothesis when we compared the context recollection parameter between the frequent versus infrequent configuration of sources, *RC*_*C1L*_ (*M* = 0.225, *SD* = 0.213) versus *RC*_*C2L*_ (*M* = 0.238, *SD* = 0.283), BF_+0_ = 0.145, *W* = 318.00. Similarly, a two-sided test provided moderate evidence for the null hypothesis when this parameter was compared between equally frequent configurations, *RC*_*C1S*_ (*M* = 0.176, *SD* = 0.234) versus *RC*_*C2S*_ (*M* = 0.213, *SD* = 0.253), BF_10_ = 0.262, *W* = 255.00. However, extreme evidence was provided for the alternative hypothesis that the response bias for the *Colour1?* question, *b*_*C1*_, was higher (*M* = 0.415, *SD* = 0.232) than for the *Colour2?* question, *b*_*C2*_ (*M* = 0.270, *SD* = 0.184), BF_+0_ = 400.365, *W* = 649.00.

In the reinstated condition, we also found moderate evidence for the null hypothesis that the context recollection parameter is equal between the frequent configuration, *RC*_*C1L*_ (*M* = 0.216, *SD* = 0.235) versus the infrequent configuration, *RC*_*C2L*_ (*M* = 0.226, *SD* = 0.290), BF_+0_ = 0.149, *W* = 178.00. In the case of the equally frequent configurations, the two-sided test yielded weak evidence for the null hypothesis, *RC*_*C1S*_ (*M* = 0.120, *SD* = 0.194) versus *RC*_*C2S*_ (*M* = 0.177, *SD* = 0.239), *BF*_*10*_ = 0.651, *W* = 96.00.

In the reinstated condition, though the colours were unequally distributed for the words printed in large font, we found only weak evidence for the alternative hypothesis that the response bias parameter *b*_*C1L*_ (*M* = 0.406, *SD* = 0.242) is larger than the *b*_*C2L*_ parameter (*M* = 0.332, *SD* = 0.251), BF_+0_ = 1.225, *W* = 370.00. Moderate evidence was provided for the null hypothesis that the response biases are equal for equally probable colours presented in small font, *b*_*C1S*_ (*M* = 0.333, *SD* = 0.239) versus *b*_*C2S*_ (*M* = 0.344, *SD* = 0.247), BF_10_ = 0.181, *W* = 287.00.

In the case of comparisons between the neutral and reinstated conditions, all Bayes factors provided weak or moderate support for the null hypotheses (0.244 < BF_10_ < 0.507, Bayesian Mann–Whitney *U* tests).

### Discussion

The results of Experiment 1 provided support for the base-rate neglect hypothesis over the base-rate dependency hypothesis. Analyses on the descriptive measures indicated that the base-rates experienced during the study do not influence accurate memory for context. Similarly, no differences between context recollection parameters between frequent and infrequent configurations were observed in multinomial analyses. However, evidence supported the hypothesis that the participants guess the colour in accordance with the base-rate. Evidence for probability-matching of guessing strategy was stronger in the neutral condition than in the reinstated condition, probably because the task of matching to Colour 1 / Colour 2 ratio was an easier task than matching strategy to a more complex colour and font size configuration. No support was found in favour of the hypothesis that reinstating one of the correlated features cues the retrieval of the other feature facilitating base-rate dependency. Initial analyses comparing accurate CARs between Colour 1 and Colour 2 items revealed that the less frequent colour is more accurately attributed to the test item than the more frequent colour. This suggests a kind of fan effect (Anderson 1974), in which it is easier to retrieve information when it has fewer associations. Item-to-context associations may be stronger for Colour 1 because it has a smaller fan of connections with individual items than Colour 2. However, such a fan effect should not refer to context-to-context associations, since these connections are learned through repeated exposure to the same features (colour and size) that do not form unique pairs.

The conclusion that our memory ignores base-rate information seems premature from the above results. Although most of these results supported the base-rate neglect hypothesis, when context recollection parameters were compared between equally frequent configurations, weak to moderate evidence for the null hypothesis indicated that base-rates are not completely disregarded. In the remainder of this article, we present two more experiments with a different procedure intended to enhance the encoding of an integrated episodic trace. Binding contextual features with each other and with item information to create an episodic trace is crucial for the reconstructive processes during a memory test. It is possible, however, that binding size with colour information was not effective in Experiment 1, since it is difficult to find any semantic association between these features. In Experiment 2, we purposely used elements that are easier to bind and we explicitly asked the participants to try to create an integrated image of an object having particular features. Such an instruction can facilitate context reinstatement effects (e.g., Hanczakowski et al. 2014; Hockley 2008).

## Experiment 2: Memory for unequally distributed extrinsic versus intrinsic features

In the second experiment, we examined how retrieving one feature associated with an object depends on the base-rate of objects’ feature configurations. The feature of interest (colour) was presented either as an intrinsic property of the object or as a distinct element. In the latter case, it can be argued that the participants encoded the configurations of items, similar to an associative recognition task but with repeating pairings.

The participants were required to memorise *colour-object-location* triplets (cf. Horner and Burgess 2013). In one condition, the colour information was represented by a separate word—the name of the colour, and, in another condition, it was represented as a font colour. Particular colours were frequently paired with one of two locations and infrequently with the other. Therefore, in this experiment, we examined whether the base-rate of the colour/location configuration can influence the retrieval of the colour of an object. We assumed that if such a dependency exists it should be more pronounced when the object-location pairs are reinstated at test in comparison with a neutral condition when only the objects are provided as test probes. Therefore, in comparison with Experiment 1, we extended our investigation on the base-rate dependency versus the base-rate neglect hypotheses to the features that are extrinsic to the item, and we made contextual and item information binding easier and more effective.

### Participants

In this experiment, 72 undergraduates took part in exchange for course credit. Their mean age was 21.97 years (*SD* = 4.12), 8 were men. Each participant was assigned to two experimental tasks among four possible (i.e., font colour and location reinstated, colour name and location reinstated, font colour and neutral, and colour name and neutral). The two tasks assigned to a participant were prepared in two versions, differing in materials (*Blue/red and House/store* versus *Green/yellow and Forest/garden*) in order to minimize the interference between the tasks. Hence, we planned to gather 144 data sets, with the conditions being manipulated between subjects. However, due to a mistake in task assignment, a part of the participants (30) received tasks from conditions that differed in one but not the other factor (either font colour vs. name or reinstated vs. neutral condition). In order to fully preserve between-subjects design for these conditions, we had to exclude one of the two sets obtained from each of these 30 participants. Two other data sets were not recorded due to technical problems. Finally, we analysed 112 datasets: 27 in the font colour and location reinstated condition, 28 in the colour name and location reinstated condition, 28 in the font colour and neutral condition, and 29 in the colour name and neutral condition.

### Stimuli

Among Polish nouns of medium valence and frequency, medium or low arousal level, and medium or high imaginability according to the Imbir (2016) dataset, we selected 140 words. Half of them referred to objects that can occur in the blue or red colours and which can be found in a house or a store (exemplary English equivalents: *candy*, *toy*, *brush*, and *ribbon*), the other half referred to objects that can occur in green or yellow colours and which can be found in a forest or a garden (e.g., *apple*, *balloon*, *tent*, and *butterfly*). The selected 140 nouns had *M* = 6 number of letters (range: 4–8), *M* = 1.92 concreteness (range: 1.44–3.2, on a scale from 1 to 9, where 1 means high concreteness and 9 high abstractedness), *M* = 7.87 imageability (range: 7.32–8.42), and *M* = 380 frequency of appearance in the language (range: 25–1459) (Mandera et al. 2014).

### Procedure and design

**Fig. 5:**
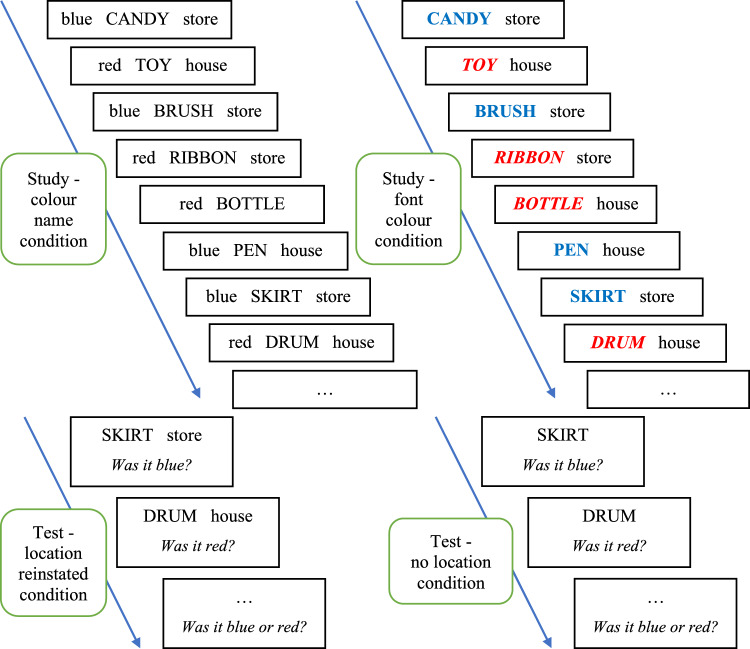
Procedure of Experiment 2. During study, the information about the colour, object, and place were provided, and colour information was represented as a word or a font. During the conjoint recognition test, the object or object-and-place were used as the cues, and the participants were asked about the colour information

The experiment was built in *OpenSesame* (Mathôt et al. 2011), and conducted online on the Jatos platform. A schematic description of the procedure is presented in Fig. 5. At study, we manipulated the way these features were represented—as a font colour in which the target word referring to an object (e.g., *apple*) was printed, or by the name of the colour paired with the target word (e.g., *green*). At test, we manipulated the reinstatement versus the absence of the word representing the location in an environment of the object that was present at study (e.g., *green-apple-garden*). The full crossing of these two between-subjects variables resulted in four experimental conditions (font colour and location reinstated; colour name and location reinstated; font colour and neutral; and colour name and neutral). In all the conditions, the frequency of a configuration (30 vs. 18 colour-location pairs) was manipulated within-subjects.

In the font colour condition, the participants were presented with 52 pairs of nouns (48 of them were targets, 2 were added as buffers at the beginning, and another 2 at the end of the study list). The first noun in each pair referred to one of the 52 objects, and the second referred to one of two places—a house or store (forest or garden in the second version) in which that object can be found. Each noun referring to an object was printed in uppercase blue or red font (green or yellow font in the second version); the words referring to places were always printed in lowercase white font.

In the colour name condition, the participants were presented with 52 triads, the second and third words in each triad referred to the object and place in the same way as in the font colour condition. However, all the words were printed in white font, and the first word in each triad was the name of one of two colours—blue or red (green or yellow in the second version). In both types of study conditions, the frequencies of colour/location configurations were not equal; among 48 targets, 30 were presented in ‘frequent’ configurations (e.g., 15 in red and house plus 15 in blue and store) and 18 in the ‘infrequent’ configurations (e.g., 9 in blue and house plus 9 in red and store).

The participants were instructed to try to remember each object with its colour and location, they were encouraged to create an image of an object in a particular colour and placed in its location. The slides were presented at a 6 s rate, with a 200 ms interstimulus interval. The stimuli were presented in *Mono* font, 38 px size; the background screen was black.

At test, 66 nouns referring to objects were presented: 48 targets and 18 distractors. In the neutral condition, a single noun was presented, that is, without the noun referring to the location. In the reinstated test condition, the targets were paired with the same location words as during the study phase. Half of the distractors were presented with one location word, the other half with the second location word. The participants were informed that their task was to answer “yes” or “no” to the question that will be presented on a particular slide. There were three types of probe questions: (a) Was this word presented in Colour 1 (with the word *Colour 1*)?; (b) Was this word presented in Colour 2 (with the word *Colour 2*)?; and (c) Was this word presented in either Colour 1 or Colour 2 (with either the word *Colour 1* or *Colour 2*)? The slides were presented in a random order, at a self-paced rate.

The software used to conduct the experiment randomly assigned colours to objects and particular (frequent or infrequent) configurations of colour and location to the participants. The probe question types assigned to the test items were counterbalanced across the test items. The sequence of task versions (*Blue/red and House/store* vs. *Green/yellow and Forest/garden*) and test condition (reinstated vs. neutral) were assigned to approximately an equal number of participants by one of the authors who distributed the links to the online experiment to the participants by email.

## Results and discussion

### Results based on descriptive measures

**Fig. 6:**
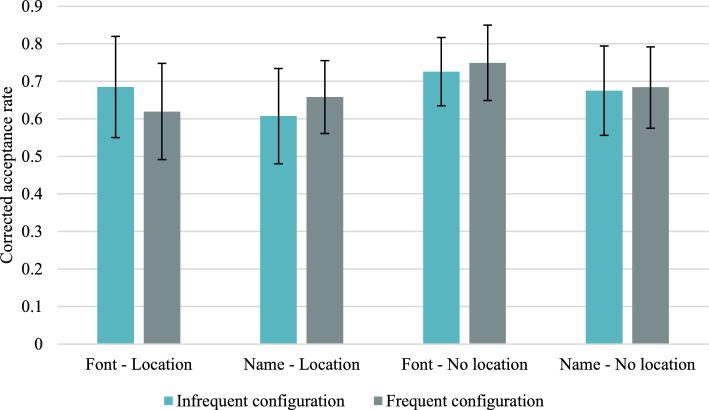
Mean corrected-for-guessing acceptance rates for accurate colour recognition for frequent versus infrequent configurations of features in Experiment 2. Error bars represent 95% credible intervals

**Fig. 7:**
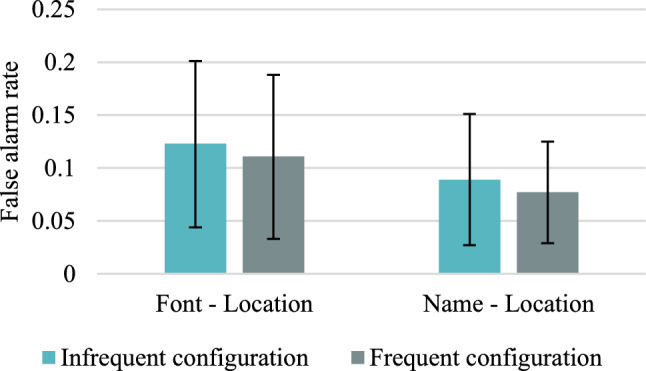
False alarm rates in Experiment 2. Error bars represent 95% credible intervals

Descriptive statistics concerning the mean acceptance rates and the mean corrected acceptance rates (CAR) for frequent versus infrequent colour/location configurations in different study and test conditions are presented in Tables 18 and 19 in “Appendix 3”. The mean CARs for accurate responses are shown in Fig. 6, and false alarms for distractors are presented in Fig. 7.

#### Within-subjects effects of base-rates

If memory is informed by the base-rates, the accurate mean CAR should be higher for the frequent configuration than the infrequent configuration, since more learning episodes should result in a stronger contextual trace or stronger context-context bindings, therefore, the one-sided alternative hypothesis is CAR_frequent_ > CAR_infrequent_. However, Bayesian Wilcoxon signed-rank test yielded strong evidence for the null hypothesis in the font colour location reinstated condition, BF_+0_ = 0.084, and moderate evidence in the colour name neutral condition, BF_+0_ = 0.206. Weak evidence for the null hypothesis was obtained in the colour name location reinstated condition, BF_+0_ = 0.442, and in the font colour neutral condition, BF_+0_ = 0.369.

In the case of false alarms, if the response bias was informed by base-rates, the participants in the reinstated condition should respond “yes” more often to distractors presented with frequent rather than infrequent location for a particular colour. Both for font colour and colour name conditions, we found moderate evidence in favour of the null hypothesis, BF_+0_ = 0.167 and BF_+0_ = 0.146, respectively. Therefore, it seems that the response bias was not informed by the base-rates.

#### Between-subjects effects of the reinstated versus the neutral test condition and of the form of colour information representation

We explored the role of representing the colour of an object as a font colour versus as a word. Table 3 presents the Bayes factors, all of which provide weak or moderate support for the null hypothesis that the form of colour representation does not affect memory performance.

**Table 3:** Results of the Bayesian Mann–Whitney *U* test for corrected acceptance rates between the font colour versus colour name study conditions

Font colour versus colour name	Frequent configuration	Infrequent configuration	Frequent configuration	Infrequent configuration
	Neutral	Neutral	Reinstated	Reinstated
Bayes factor BF_10_	0.343	0.296	0.283	0.444
Decision	Weak support for H_0_	Moderate support for H_0_	Moderate support for H_0_	Weak support for H_0_
Mann–Whitney test statistics	*W* = 348.50	*W* = 386.00	*W* = 370.50	*W* = 445.50

As in Experiment 1, we assumed that the reinstated condition in comparison with the neutral condition should result in acceptance rates that are closer to the base-rates. Therefore, for the frequent configuration, the mean CAR should be higher in the reinstated than in the neutral condition, but for the infrequent configuration, it should be lower in the reinstated than in the neutral condition. However, Table 4 shows the Bayes factors indicating evidence for the hypothesis that the neutral and the reinstated conditions do not differ in the mean CARs.

**Table 4:** Results of the Bayesian Mann–Whitney *U* test for the corrected acceptance rates between the neutral versus the reinstated test conditions

Neutral versus reinstated	Frequent configuration	Infrequent configuration	Frequent configuration	Infrequent configuration
	Font colour	Font colour	Colour name	Colour name
Bayes factor	BF_−0_ = 0.119	BF_+0_ = 0.326	BF_−0_ = 0.176	BF_+0_ = 0.611
Decision	Moderate support for H_0_	Moderate support for H_0_	Moderate support for H_0_	Weak support for H_0_
Mann–Whitney test statistics	*W* = 481.00	*W* = 382.00	*W* = 361.00	*W* = 348.50

### Results based on process measures

Table 5 presents the results of hierarchical multinomial processing tree modelling for all the experimental conditions. A good model fit was indicated by the nonsignificant test results in the font colour reinstated location condition (T1: *p* = 0.506, T2: *p* = 0.452), in the colour name reinstated location condition (T1: *p* = 0.532, T2: *p* = 0.584), in the font colour neutral condition (T1: *p* = 0.524, T2: *p* = 0.195), and in the colour name neutral condition (T1: *p* = 0.536, T2: *p* = 0.362).

**Table 5:** Group-level parameter estimates (standard deviations) and 95% BCIs of the dual-recollection multinomial model obtained in Experiment 2

Parameter	Frequent configuration	Infrequent configuration	Frequent configuration	Infrequent configuration
	Font colour	Font colour	Colour name	Colour name
Neutral condition				
*RT*	0.575 (0.065) [0.444, 0.701]	0.572 (0.061) [0.454, 0.692]	0.406 (0.056) [0.299, 0.520]	0.420 (0.064) [0.298, 0.551]
*RC*	0.585 (0.053) [0.475, 0.684]	0.482 (0.063) [0.351, 0.599]	0.599 (0.063) [0.467, 0.714]	0.548 (0.084) [0.367, 0.702]
*F*	0.176 (0.147) [0.005, 0.541]	0.243 (0.181) [0.009, 0.669]	0.227 (0.142) [0.011, 0.533]	0.271 (0.151) [0.018, 0.564]
*b*_*C1*_ = *b*_*C2*_	0.052 (0.021) [0.017, 0.098]		0.042 (0.022) [0.009, 0.095]	
*b*_*C1or2*_	0.042 (0.023) [0.007, 0.096]		0.043 (0.020) [0.011, 0.088]	
Reinstated condition				
*RT*	0.364 (0.087) [0.184, 0.534]	0.507 (0.099) [0.313, 0.707]	0.390 (0.051) [0.290, 0.492]	0.433 (0.069) [0.289, 0.564]
*RC*	0.457 (0.090) [0.272, 0.624]	0.461 (0.113) [0.223, 0.668]	0.492 (0.069) [0.343, 0.616]	0.339 (0.096) [0.141, 0.519]
*F*	0.268 (0.178) [0.014, 0.683]	0.193 (0.167) [0.005, 0.638]	0.177 (0.117) [0.008, 0.430]	0.255 (0.140) [0.018, 0.530]
*b*_*Cf*_	0.065 (0.034) [0.013, 0.141]		0.050 (0.026) [0.010, 0.111]	
*b*_*Ci*_	0.065 (0.037) [0.011, 0.151]		0.051 (0.027) [0.009, 0.113]	
*b*_*Cf or i*_	0.053 (0.038) [0.005, 0.144]		0.054 (0.032) [0.008, 0.130]	

The italicized symbols are the parameters of the dual-recollection multinomial model: *RT* = target recollection, *RC* = context recollection, *F* = familiarity, and *b* = response bias depending on the probe question

The effects of within-subjects manipulation of the frequency of the colour/location configuration are shown in Table 6. The credible intervals included 0 for all the differences of the parameter estimates, indicating no substantial effect.

**Table 6:** Differences in the mean dual-recollection multinomial model parameters and 95% BCIs between the frequent and the infrequent configurations

Parameter	Neutral	Reinstated	Neutral	Reinstated
(Frequent–Infrequent)	Font colour	Font colour	Colour name	Colour name
Δ*RT*	0.002 [− 0.161, 0.166]	− 0.143 [− 0.396, 0.106]	− 0.012 [− 0.172, 0.150]	− 0.041 [− 0.196, 0.125]
Δ*RC*	0.102 [− 0.036, 0.244]	0.003 [− 0.216, 0.242]	0.046 [− 0.105, 0.211]	0.153 [− 0.041, 0.361]
Δ*F*	− 0.069 [− 0.544, 0.383]	0.090 [− 0.397, 0.562]	− 0.042 [− 0.419, 0.356]	− 0.081 [− 0.423, 0.272]
Δ*b*	–	− 0.001 [− 0.09, 0.082]	–	− 0.001 [− 0.073, 0.068]

Δ*RT* = the difference in the target recollection parameter estimates, Δ*RC* = the difference in the context recollection parameter estimates, Δ*F* = the difference in the familiarity parameter estimates, and Δ*b* = the difference in the response bias parameter estimates

Between-subjects manipulation of the reinstated versus the neutral test condition demonstrated one substantial but unexpected effect—the target recollection parameter was higher for the neutral than the reinstated test condition in the case of the frequent configuration in the font colour condition (see Table 7).

**Table 7:** Differences in the mean dual-recollection multinomial model parameters and 95% BCIs between the neutral and the reinstated location conditions

Parameter	Frequent configuration	Infrequent configuration	Frequent configuration	Infrequent configuration
Neutral − Reinstated	Font colour	Font colour	Colour name	Colour name
Δ*RT*	**0.219** **[0.007, 0.441]**	0.072 [− 0.159, 0.307]	0.021 [− 0.124, 0.171]	− 0.006 [− 0.183, 0.183]
Δ*RC*	0.135 [− 0.071, 0.356]	0.038 [− 0.216, 0.311]	0.124 [− 0.060, 0.309]	0.230 [− 0.025, 0.477]
Δ*F*	− 0.073 [− 0.536, 0.401]	0.071 [− 0.438, 0.577]	0.059 [− 0.299, 0.438]	0.020 [− 0.379, 0.424]

Δ*RT* = the difference in the target recollection parameter estimates, Δ*RC* = the difference in the context recollection parameter estimates, and Δ*F* = the difference in the familiarity parameter estimates

The difference when the credibility interval does not overlap with 0 is printed in bold font

The results of between-subjects manipulation of the colour presentation form are shown in Table 8. The only credibility interval not overlapping with 0 indicated that the target recollection parameter was substantially higher for the font colour than for the colour name condition in the case of the frequent configuration neutral condition.

**Table 8:** Differences in the mean dual-recollection multinomial model parameters and 95% BCIs between the colour information presented as a font colour versus as a colour name

Parameter	Frequent configuration	Infrequent configuration	Frequent configuration	Infrequent configuration
Font Colour − Colour name	Neutral	Neutral	Reinstated	Reinstated
Δ*RT*	**0.174** **[0.001, 0.344]**	0.150 [− 0.028, 0.322]	− 0.035 [− 0.238, 0.157]	0.066 [− 0.174, 0.308]
Δ*RC*	− 0.014 [− 0.182, 0.159]	− 0.063 [− 0.272, 0.162]	− 0.043 [− 0.273, 0.177]	0.110 [− 0.193, 0.399]
Δ*F*	− 0.051 [− 0.441, 0.390]	− 0.025 [− 0.441, 0.481]	0.084 [− 0.294, 0.529]	− 0.074 [− 0.441, 0.408]

Δ*RT* = the difference in the target recollection parameter estimates, Δ*RC* = the difference in the context recollection parameter estimates, and Δ*F* = the difference in the familiarity parameter estimates

The difference when the credibility interval does not overlap with 0 is printed in bold font

#### Bayesian analyses of the hypotheses about context recollection and response bias

For the purposes of Bayes factor analyses, we calculated the independent estimates of parameters for each participant using the maximum likelihood fitting method implemented in the *multiTree* software (Moshagen 2010). The results of analyses conducted using this method and based on aggregated data are presented in "Appendix 4". Here, we focus on the hypotheses concerning the context recollection parameter.

##### Within-subjects effects of base-rates on the context recollection and the response bias parameters

If memory is informed by the base-rates, the context recollection parameter should be higher for the frequent than for the infrequent configurations, therefore, the one-sided alternative hypothesis is *RC*_frequent_ > *RC*_infrequent_. As Table 9 shows, moderate support for the alternative hypothesis was found in the font colour neutral condition, and moderate support in favour of the null hypothesis was obtained in the font colour reinstated condition. In the case of the colour name conditions, the Bayes factors were inconclusive. When it came to the response bias parameter, the Bayes factors indicated moderate evidence for the null hypothesis, both in the font colour reinstated condition, BF_+0_ = 0.112, *W* = 38.00, and in the colour name reinstated condition, BF_+0_ = 0.243, *W* = 27.00. Therefore, the response bias seems to be not informed by the base-rates.

**Table 9:** Results of the Bayesian Wilcoxon Signed-Rank test comparisons of the context recollection parameter for the infrequent versus the frequent configurations

Infrequent versus frequent	Neutral	Reinstated	Neutral	Reinstated
	Font colour	Font colour	Colour name	Colour name
Bayes factor BF_−0_	3.918	0.233	0.680	1.302
Decision	Moderate support for H_1_	Moderate support for H_0_	Weak support for H_0_	Weak support for H_1_
Wilcoxon test statistics	*W* = 118.50	*W* = 145.00	*W* = 164.50	*W* = 138.00

##### Between-subjects effects of the reinstated versus the neutral test condition and of the form of colour information representation

As in previous analyses, the direction of the alternative hypothesis about the effects of reinstatement manipulation depended on the frequency of the colour/location configuration. As shown in Table 10, in most conditions, the data favoured the null hypothesis about the lack of any differences between the test conditions; Bayes factor was inconclusive only in the infrequent configuration colour name condition.

**Table 10:** Results of the Bayesian Mann–Whitney *U* test comparisons of the context recollection parameter for the neutral versus the reinstated test conditions

Neutral versus reinstated	Frequent configuration	Infrequent configuration	Frequent configuration	Infrequent configuration
	Font colour	Font colour	Colour name	Colour name
Bayes factor	BF_−0_ = 0.203	BF_+0_ = 0.205	BF_−0_ = 0.156	BF_+0_ = 1.117
Decision	Moderate support for H_0_	Moderate support for H_0_	Moderate support for H_0_	Weak support for H_+_
Mann–Whitney test statistics	*W* = 421.50	*W* = 356.00	*W* = 332.50	*W* = 316.00

Finally, the context recollection parameter was compared between the font colour and the colour name conditions. As demonstrated in Table 11, the Bayes factors indicated weak or moderate support for the null hypothesis.

**Table 11:** Results of the Bayesian Mann–Whitney *U* test comparisons of the context recollection parameter for the font colour versus the colour name study conditions

Font colour versus colour name	Frequent configuration	Infrequent configuration	Frequent configuration	Infrequent configuration
	Neutral	Neutral	Reinstated	Reinstated
Bayes factor BF_10_	0.326	0.419	0.276	0.367
Decision	Moderate support for H_0_	Weak support for H_0_	Moderate support for H_0_	Weak support for H_0_
Mann–Whitney test statistics	*W* = 427.50	*W* = 464.00	*W* = 379.00	*W* = 427.00

## Experiment 3: The role of the magnitude of base-rate difference: A follow-up study

The decision-making literature suggests that under some conditions people are sensitive to base-rates in their probability judgments. One of these conditions is when base-rates are made more extreme (Koehler 1996). In the final experiment, we manipulated the magnitude of the disproportion in base-rates between feature configurations to see if we would observe an effect of high disproportion on memory and response biases. In doing so, we also hoped that the experiment would help clarify the inconsistency of the results of Experiments 1 and 2 regarding the sensitivity of response bias to the base-rate.^2^

In Experiment 1 we obtained strong support for the hypothesis that the participants match their responses with the experienced frequency of contexts (e.g., Bayen and Kuhlmann 2011; Kuhlmannn et al. 2012; Wulff et al. 2021). However, in Experiment 2, no difference in the response bias was found when the distractors were presented with frequent versus infrequent locations, and the Bayes factor indicated moderate evidence for the null hypothesis. This discrepancy may be due to a more salient difference in the proportions of feature configurations in Experiment 1 than in Experiment 2. In Experiment 1, Colour 1 was four times more probable than Colour 2 for a large font (36:9), but in Experiment 2, however, Colour 1 was only about 1.7 times more probable than Colour 2 (30:18); hence, it was more difficult for the participants to notice this difference in the base-rate. An alternative explanation is that participants in Experiment 2 could be aware of the base-rates, but they did not rely on them in guessing strategy due to their metamemory judgment of good memory (indeed their memory was much better in Experiment 2 than in Experiment 1), which did not require notable adjustment.

In Experiment 3, we used generally the same materials and procedure as in the font colour location reinstated condition of Experiment 2, but we varied the base-rates and increased the number of stimuli. Higher number of items in Experiment 3 (60 targets and 30 distractors) in comparison with Experiment 2 (48 targets and 18 distractors) should decrease participants’ confidence in memory judgments giving more room for guessing. Concerning the base-rates manipulation, we compared a more salient disproportion with a less salient disproportion of features in particular locations, that is, in the *high disproportion condition*, one colour was four times more probable than the other in a given location (48:12), and in the *low disproportion condition*, one colour was 1.5 times more probable than the other (36:24). These differences in base-rates are similar to those in Experiment 1 and Experiment 2, where the response bias effect was and was not observed, respectively.

Moreover, to minimize instances in which participants automatically respond to probe questions, mistakenly responding to a different question than the one actually asked, we distinguished the type of question with colour font corresponding to the content of the question (e.g., “Was this word presented in GREEN?” presented in green font colour). This procedural change can improve context reinstatement when the probe question matches the target context (Symeonidou and Kuhlmann 2021).

### Participants

In this experiment, 66 first year part-time psychology students took part in exchange for course credit. Their mean age was 23.00 years (*SD* = 5.91), 12 were men. The number of participants per condition was similar as in Experiment 2. Each participant was randomly assigned to the high disproportion condition (n = 33) or the low disproportion condition (n = 33). Two participants who failed to follow the instructions were excluded, one from each group.

### Stimuli

From the same dataset (Imbir 2016) as in Experiments 1 and 2 we selected 94 nouns. They referred to objects that can occur in green or yellow colours and which can be found in a forest or a garden. The selected nouns had *M* = 5.9 number of letters (range: 4–8), *M* = 1.9 concreteness (range: 1.48–3.2), *M* = 7.92 imageability (range: 7.54–8.42), and *M* = 354 frequency of appearance in the language (range: 36–1357).

### Procedure and design

The participants were examined at individual workstations in the University Lab. The presentation of the stimuli and the response recording were conducted using the E-Prime 2.0 program.

At study, the participants were presented with 64 pairs of nouns (60 of them were targets, 2 were added as buffers at the beginning, and another 2 at the end of the study list). The first noun in each pair referred to one of the 64 objects, and the second referred to one of two places—a forest or garden. Each noun referring to an object was printed in the uppercase green or yellow font; the words referring to places were always printed in lowercase white font. Buffers at the beginning and at the end of the study list were presented in the more frequent colour-place configuration. As in Experiment 2, the participants were instructed to try to remember each object with its font colour and location and to create an image to help in remembering. The slides were displayed at a 6 s rate, with a 200 ms interstimulus interval. The stimuli were presented in *Arial* font, 48 pts size; the background screen was black.

The frequency of configurations (frequent vs. infrequent colour-location pairs) was manipulated within subjects, but the magnitude of disproportion in base-rates was manipulated between subjects. In the high disproportion condition it was: 12 infrequent colour-location pairs (e.g., 6 green and garden plus 6 yellow and forest) and 48 frequent pairs (e.g., 24 yellow and garden plus 24 green and forest), and in the low disproportion condition it was: 24 infrequent pairs (e.g., 12 green and garden plus 12 yellow and forest) and 36 frequent pairs (e.g., 18 yellow and garden plus 18 green and forest).^3^

At test, 90 nouns referring to objects were presented: 60 targets and 30 distractors. As in the reinstated condition of Experiment 2, the targets were paired with the same location words as during the study phase. Half of the distractors were presented with one location word, the other half with the second location word. The three types of probe questions were: (a) Was this word presented in COLOUR 1?; (b) Was this word presented in COLOUR 2 ?; and (c) Was this word presented in either COLOUR 1 or COLOUR 2 ? The questions were presented in a font corresponding to the content of the question: green, yellow or white. The slides were presented in a random order, at a self-paced rate. The assignment of colours (green, yellow) to frequent or infrequent configurations and probe question types to the test items were counterbalanced across participants.

## Results and discussion

### Results based on descriptive measures

**Fig. 8:**
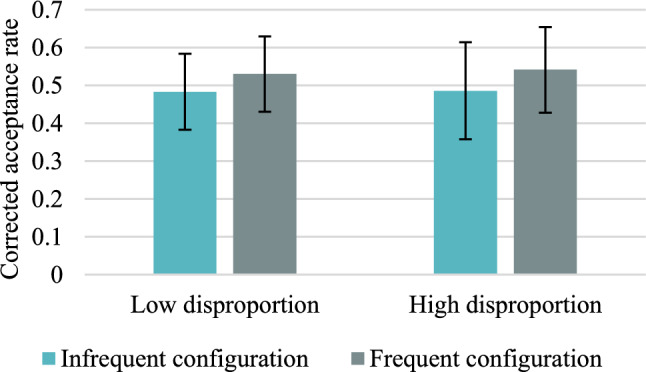
Mean corrected-for-guessing acceptance rates for accurate colour recognition for frequent versus infrequent configurations of features in Experiment 3. Error bars represent 95% credible intervals

**Fig. 9:**
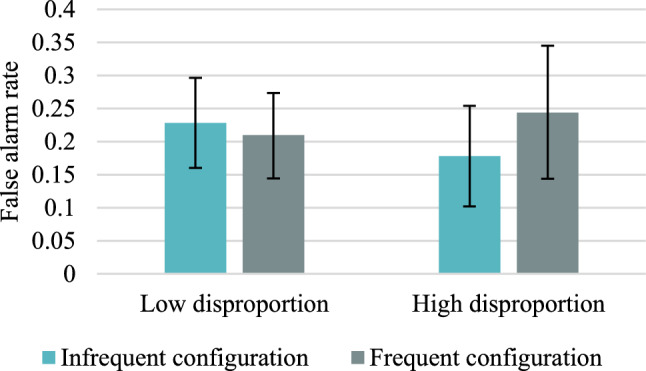
False alarm rates in Experiment 3. Error bars represent 95% credible intervals

Descriptive statistics concerning the mean acceptance rates and the mean CARs for frequent versus infrequent colour/location configurations in high versus low disproportion conditions are presented in Tables 22 and 23 in "Appendix 5". The mean CARs for accurate responses are shown in Fig. 8, and false alarms for distractors are presented in Fig. 9.

#### Within-subjects effects of base-rates

As in previous experiments, the one-sided alternative hypothesis is CAR_frequent_ > CAR_infrequent_. In the low disproportion condition, the Bayesian Wilcoxon signed-rank test yielded weak evidence for the null hypothesis, BF_+0_ = 0.662. Similarly, in the high disproportion condition, the Bayesian *t* test yielded weak support for the null hypothesis, BF_+0_ = 0.480. Therefore, on the descriptive level of analysis, participants’ memory seems to be not dependent on the base-rates, even when there is a high level of disproportion between frequent and infrequent configuration of features. This result replicates observations from Experiments 1 and 2.

In the case of false alarms, the Bayesian Wilcoxon signed-rank test indicated weak evidence in favour of the alternative hypothesis, BF_+0_ = 2.080, in the high disproportion condition, but moderate in favour of the null hypothesis, BF_+0_ = 0.117, in the low disproportion condition. Therefore, as in Experiment 2, the response bias was not informed by base-rates in the low disproportion condition, but in the high disproportion condition it is 2 times more probable that response bias is affected by the base-rates than that it is not affected.

#### Between-subjects effects of the magnitude of base-rate difference

Table 12 shows the Bayes factors, all of which provide weak or moderate support for the null hypothesis that the magnitude of the disproportion does not affect accurate target recognition or false alarms to distractors.

**Table 12:** Results of the Bayesian Mann–Whitney *U* test for corrected acceptance rates between the high versus low disproportion conditions

High versus low disproportion	Frequent configuration	Infrequent configuration	Frequent configuration	Infrequent configuration
	Hits	Hits	False alarms	False alarms
Bayes factor BF_10_	0.261	0.272	0.268	0.396
Decision	Moderate support for H_0_	Moderate support for H_0_	Moderate support for H_0_	Weak support for H_0_
Mann–Whitney test statistics	*W* = 526.00	*W* = 523.00	*W* = 500.50	*W* = 420.50

### Results based on process measures

Table 13 presents the results of hierarchical multinomial processing tree modelling for both experimental conditions. An acceptable model fit was indicated by the nonsignificant test results in the high disproportion condition (T1: *p* = 0.494, T2: *p* = 0.342), and in the low disproportion condition (T1: *p* = 0.385, T2: *p* = 0.055).

**Table 13:** Group-level parameter estimates (standard deviations) and 95% BCIs of the dual-recollection multinomial model obtained in Experiment 3

Parameter	Frequent configuration	Infrequent configuration	Frequent configuration	Infrequent configuration
	High disproportion	High disproportion	Low disproportion	Low disproportion
*RT*	0.460 (0.055) [0.345, 0.562]	0.507 (0.070) [0.358, 0.639]	0.442 (0.060) [0.321, 0.556]	0.383 (0.059) [0.262, 0.497]
*RC*	0.436 (0.058) [0.314, 0.543]	0.202 (0.070) [0.053, 0.331]	0.315 (0.066) [0.179, 0.438]	0.291 (0.068) [0.150, 0.418]
*F*	0.174 (0.098) [0.012, 0.371]	0.432 (0.225) [0.038, 0.872]	0.107 (0.082) [0.004, 0.300]	0.142 (0.111) [0.005, 0.409]
*b*_*Cf*_	0.163 (0.057) [0.068, 0.289]		0.190 (0.036) [0.122, 0.262]	
*b*_*Ci*_	0.120 (0.042) [0.048, 0.214]		0.210 (0.037) [0.139, 0.286]	
*b*_*Cf or i*_	0.057 (0.027) [0.014, 0.119]		0.140 (0.035) [0.077, 0.215]	

The italicized symbols are the parameters of the dual-recollection multinomial model: *RT* = target recollection, *RC* = context recollection, *F* = familiarity, and *b* = response bias depending on the probe question

In the high disproportion condition, the mean difference in context recollection parameters Δ*RC* between frequent versus infrequent configurations (*RC*_*Cf*_ − *RC*_*Ci*_) was *M* = 0.240, with the credibility interval of the difference [0.069, 0.415] not overlapping with 0, indicating a substantial effect. In the low disproportion condition, no such effect was found for Δ*RC*, *M* = 0.024 with 95% CI [− 0.150, 0.197]. This result indicates that the context recollection becomes sensitive to the base rate when the disproportion between configurations is made very salient. It is worth noting that the target recollection parameter was numerically higher for the infrequent configuration than for the frequent configuration, indicating a dissociation between the recollection processes.

For the difference between response bias parameters (*b*_*Cf*_ − *b*_*Ci*_) no substantial effects were detected both in the high disproportion condition, *M* = 0.043 with 95% CI [− 0.052, 0.146], and the low disproportion condition, *M* =  − 0.02 with 95% CI [− 0.105, 0.066].

Between-subjects manipulation of the magnitude of disproportion in the base-rate yielded no substantial effect for the context recollection parameter, for the frequent configuration of features (*RC*_*Cf* high_ − *RC*_*Cf* low_), *M* = 0.119 with 95% CI [− 0.054, 0.290], and for the infrequent configuration of features (*RC*_*Ci* high_ − *RC*_*Ci* low_), *M* =  − 0.091 with 95% CI [− 0.285, 0.100].

#### Bayesian analyses of the hypotheses about context recollection and response bias

For the purposes of Bayes factor analyses, we calculated the independent estimates of parameters for each participant using the maximum likelihood fitting method. The results of the analyses using this method, based on the aggregated data, are presented in Table 25 in "Appendix 6".

##### Within-subjects effects of base-rates on the context recollection and the response bias

For the context recollection parameter, the Bayesian Wilcoxon signed-rank test indicated strong support for the alternative hypothesis in the high disproportion condition, BF_+0_ = 11.263, *W* = 362.00, but moderate support in favour of the null hypothesis in the low disproportion condition, BF_+0_ = 0.196, *W* = 254.00.

In similar vein, when it came to the response bias parameter, the Bayes factors indicated moderate evidence for the alternative hypothesis in the high disproportion condition, BF_+0_ = 5.624, *W* = 206.00, but moderate evidence for the null hypothesis in the low disproportion condition, BF_+0_ = 0.148, *W* = 178.00. Although the difference between the response bias parameters were not significant, Bayesian analyses supported the prediction that more salient differences in base rates influence participants’ response strategy.

##### Between-subjects effects of the magnitude of disproportion on the context recollection and the response bias parameters

The Bayesian Mann–Whitney *U* test favoured the null hypothesis about the lack of any differences between the high versus low disproportion conditions. For the context recollection parameter, Bayes factors weakly or moderately supported the null hypothesis, for frequent configurations, BF_10_ = 0.407, *W* = 601.00, and infrequent configurations of features, BF_10_ = 0.314, *W* = 452.00. For the response bias parameters, the Bayes factors also indicated weak or moderate support for the null hypothesis, in the case of guessing “yes” to frequent configuration, BF_10_ = 0.271, *W* = 505.00, and in the case of guessing “yes” to infrequent configuration, BF_10_ = 0.375, *W* = 419.00.

In sum, in Experiment 3 we found a significant difference in the context recollection parameter between frequent and infrequent configurations when differences in the base rates were high, but not when they were less salient. Bayes factor indicated strong support for base-rate dependency under high disproportion condition. Some support, although less unequivocal, was also provided to the hypothesis of the role of salient difference in base-rates on response bias.

## General discussion

In three experiments, using the Bayesian inference, we weighted the evidence in favour of the base-rate dependency versus the base-rate neglect hypotheses in memory for the correlated features. The first two studies and one condition of the third experiment, supported the base-rate neglect hypothesis. In Experiment 1, the memory for the font colour did not depend on whether this feature was equally or unequally distributed among the words printed in large versus small font. We found no support for such a dependency even when the correlated feature was reinstated at retrieval. This result was somewhat surprising, taking into account the stochastic dependence reported in many studies on multidimensional source memory (e.g., Arnold et al. 2019; Boywitt and Meiser 2012; Meiser 2014; Meiser and Bröder 2002) that was interpreted as indicating context-context binding in episodic memory. However, our results are consistent with the suggestions in literature that a stochastic dependence stems from item-context rather than from context-context binding (e.g., Hicks and Starns 2016; Starns and Hicks 2005; Vogt and Bröder 2007). Therefore, providing one contextual feature (font size) was probably incapable of assisting the retrieval of another contextual feature (font colour), since these context dimensions are not bound directly to one another (Hicks and Starns 2016). Another possibility is that context-context bindings do exist, but the effects of mutual cueing were undetectable since the contextual feature probed in memory test was very poorly encoded. In Experiment 1, the non-overlapping with 0 credibility intervals of all context recollection parameters for colours indicated that participants did remember colours, however, the *RC* values were relatively low, ranging from 0.046 to 0.219. Nevertheless, this interpretation is no longer convincing when we consider the results of Experiment 2, in which the *RC* values were much higher, ranging from 0.339 to 0.599, and of the low disproportion condition in Experiment 3, with the *RC* values from 0.291 to 0.315, where support for the base-rate dependency was not found either.

Better memory for context in Experiments 2 and 3 was probably achieved because we asked the participants to create integrated images of the studied elements. In these experiments, we also tested a different form of representing features of the to-be-remembered episodes. In some of the experimental conditions, we used triplets of distinct elements representing the colour, object, and location. Following the studies on pattern completion, which suggested that the event elements are integrated into the holistic representations (e.g., Horner and Burgess 2013; Horner et al. 2015), we expected the more frequent pairings to be more effectively encoded into an episodic trace than the less frequent pairings, and that providing one of the elements at test will effectively cue another element—the stronger the binding between elements, the more effective the cueing will be. This was expected for contextual details represented by font colour, and even more so for features represented by separate items. However, in Experiment 2, we found that a manipulation of the colour/location configuration frequency did not affect memory for colour information, both when colour was represented as an intrinsic feature (font colour) and as a separate element (colour name). Instead, the Bayesian analyses supported the null hypothesis, indicating the lack of base-rate dependency. These results may be interpreted as suggesting that events are not encoded holistically (cf. Trinkler et al. 2006), at least when they do not comprise of unique triplets of elements, but two elements (colour and location) of these triplets are shared by multiple objects. Binding effects would probably be present for unique events, that is, such that are represented by specific elements.

Experiment 3 was designed with the goal of examining the role of extreme disproportion in base-rates on context memory and response bias. When there were 4 times as many targets in frequent than infrequent feature configurations, and one of the features was reinstated at test, we did find significantly higher context recollection for frequently presented than infrequently presented targets. It is noteworthy that this effect was not detected at the behavioural level, probably because context recollection and target recollection processes operated in an incompatible way. When the disproportion between base-rates was not so salient, and all other elements of the procedure and materials were the same, we again obtained support for the base-rate neglect hypothesis. The result observed in the high disproportion condition shows that under certain conditions, context recollection is sensitive to the base-rate. Similar boundary conditions for the base-rate neglect phenomenon have been found in the research on human probability judgements (Koehler 1996).

In our research, following the recent dual-recollection interpretation by Brainerd et al. (2022a), we assumed that contextual features are encoded into a contextual trace and conscious reinstatement of this trace proceeds as a phenomenon of context recollection. In one of the experiments validating the dual-recollection model, Brainerd et al. (2015, Experiment 2) presented smaller versus larger groups of semantically related words during a study phase. A manipulation of the number of related items influenced the estimates of the context recollection contribution to memory performance but did not affect the target recollection or familiarity contribution. This result prompted us to assume that context recollection is sensitive to the number of occurrences of particular contextual features and that frequent feature-feature pairings will result in stronger contextual representation than infrequent pairings—reflecting the base-rates of features. However, in our experiments, standard comparisons of the context recollection parameter estimates between the conditions differing in the base-rates of the features indicated substantial effects only when extreme differences in base-rates were used. Similarly, the Bayesian analyses, with the exception of the high disproportion condition in Experiment 3, did not support the base-rate sensitivity hypothesis for the context recollection parameter. In most conditions, we found weak or moderate evidence in favour of the null hypothesis. It seems that the effects of the number of items sharing a contextual feature on context recollection are confined to the high disproportions in base-rates and to the semantic features and are harder to obtain for low or moderate disproportions and perceptual features such as colour.

Concerning the response bias sensitivity to the base-rates, we obtained strong (Experiment 1) or moderate (the high disproportion condition of Experiment 3) support for the hypothesis that the participants match their responses with the experienced frequency of contexts (e.g., Bayen and Kuhlmann 2011; Kuhlmannn et al. 2012; Wulff et al. 2021). This effect was limited to conditions with a salient difference in the base-rates and disappeared when this disproportion was not so striking (Experiment 2 and the low disproportion condition of Experiment 3).

In our research, we did not assume that the base-rate dependence requires an intentional or explicit learning of the base-rates; as a matter of fact, there is no reason to assume that base-rate sensitivity cannot be acquired from implicit learning (cf. Wismer and Bohil 2017). Moreover, at least in Experiment 1, the participants were instructed at study that one colour font is more frequent in a particular font size than in another, and they used this knowledge to inform their guessing strategy, in spite of the fact that they neglected the base-rates in their memory for context.

To conclude this article, our findings supported the hypothesis that context memory is not sufficiently informed by the frequency of the feature pairings and is prone to base-rate neglect, at least when differences in the base-rates are not extreme. The conditional probability of one feature given another feature is not sufficiently reflected in the strength of the contextual memory trace. This memory insensitivity to the structure of real-world events is at odds with the observations of Anderson and Schooler (1991), but it resembles deep distortions; a family of memory biases (overdistribution, super-overdistribution, non-additivity, and impossible conjunctions) that violate some axioms and rules of classical probability (Brainerd 2021, 2022). In the case of base-rate neglect in memory for context, the essential standard of the Bayes’ theorem seems to be violated (Lu and Nieznański 2020). Further research is needed to define boundary conditions for the base-rate neglect in memory for contextual features. Our experiments indicated that such conditions can be created by using salient disproportion in base-rates simultaneously with facilitating feature binding. It would be worth investigating whether base-rate dependency would be present for ‘integral’ contextual features, in which changes in one dimension cannot be ignored when attention is paid to another dimension^4^ (Garner 1974). Previous research has also suggested that base-rate dependency for context recollection can be observed when contexts are defined by groups of semantically related words (Brainerd et al. 2015).

## Data Availability

Raw data can be accessed online at: https://osf.io/sz4vp/.

## Appendix 1: Results of descriptive measures used in Experiment 1

See Tables 14 and 15.

**Table 14:** Mean acceptance probabilities depending on the probe type in Experiment 1

	C1?	C2?	C1or 2?
	*M* (*SD*)	*M* (*SD*)	*M* (*SD*)
Neutral condition			
C1L (n = 36)	**0.692 (0.173)**	0.394 (0.185)	**0.654 (0.191)**
C1S (n = 18)	**0.621 (0.233)**	0.388 (0.231)	**0.688 (0.211)**
C2L (n = 9)	0.475 (0.310)	**0.617 (0.298)**	**0.775 (0.255)**
C2S (n = 18)	0.471 (0.256)	**0.588 (0.207)**	**0.637 (0.220)**
New (n = 42)	0.441 (0.265)	0.279 (0.208)	0.230 (0.140)
Reinstated condition			
C1L (n = 36)	**0.691 (0.158)**	0.437 (0.209)	**0.698 (0.221)**
C1S (n = 18)	**0.554 (0.252)**	0.518 (0.254)	**0.667 (0.242)**
C2L (n = 9)	0.550 (0.344)	**0.595 (0.295)**	**0.784 (0.251)**
C2S (n = 18)	0.482 (0.225)	**0.667 (0.272)**	**0.635 (0.269)**
NewL (n = 21)	0.425 (0.277)	0.340 (0.268)	0.266 (0.241)
NewS (n = 21)	0.347 (0.260)	0.336 (0.259)	0.301 (0.235)

Results printed in boldface indicate correct responses. C1, C2 denote Colour 1, Colour 2, respectively; L, S denote large font, small font, respectively

**Table 15:** Mean corrected acceptance probabilities for colour recognition in Experiment 1

	C1?	C2?	C1or2?
	*M* (*SD*)	*M* (*SD*)	*M* (*SD*)
Neutral condition			
C1L (n = 36)	**0.251 (0.300)**	0.115 (0.203)	**0.424 (0.197)**
C1S (n = 18)	**0.180 (0.306)**	0.109 (0.278)	**0.457 (0.253)**
C2L (n = 9)	0.034 (0.414)	**0.338 (0.357)**	**0.545 (0.252)**
C2S (n = 18)	0.230 (0.321)	**0.309 (0.275)**	**0.407 (0.232)**
Reinstated condition			
C1L (n = 36)	**0.267 (0.350)**	0.097 (0.248)	**0.432 (0.303)**
C1S (n = 18)	**0.207 (0.296)**	0.182 (0.291)	**0.365 (0.289)**
C2L (n = 9)	0.125 (0.349)	**0.255 (0.424)**	**0.517 (0.321)**
C2S (n = 18)	0.135 (0.273)	**0.331 (0.356)**	**0.334 (0.365)**

Results printed in boldface indicate correct responses

## Appendix 2: Results of multinomial modelling analyses using maximum likelihood methods for Experiment 1

The probabilities of elementary cognitive states, which are represented by the model parameters, were estimated by applying maximum likelihood procedures. Traditionally, multinomial processing tree models are fitted using data aggregated across participants and items (Table 16). Such analyses are conducted under the homogeneity assumption that the individual frequencies are identically distributed (Smith and Batchelder 2008). However, this assumption is usually violated, therefore, in the main text of this paper, results based on more sophisticated Bayesian hierarchical analyses that explicitly account for the heterogeneity of participants are provided. The hypotheses were tested with the log-likelihood ratio statistic (*G*^2^), which is distributed asymptotically as a χ^2^ distribution. At α level of 0.05, *G*^2^(1) = 3.84 indicates a critical value. The computations were carried out with the multiTree computer program (Moshagen 2010). In Experiment 1, sensitivity power analyses ensured a high test power (1 – β = 0.95) for the parameter comparisons across the conditions. With α = 0.05 and the number of responses across participants ranging from 4551 in the reinstated condition to 4920 in the neutral condition, small effect sizes ranging from *w* = 0.051 to 0.053 were detectable (analyses computed with G*Power 3; Faul et al. 2007). The parameter estimates based on the aggregated data are presented in Table 17.

**Table 16:** Aggregated frequencies of responses in Experiment 1

Question probe	C1?		C2?		C1or2?	
Response	Yes	No	Yes	No	Yes	No
Neutral condition (*N* = 40)						
C1L (n = 36)	332	148	189	291	314	166
C1S (n = 18)	149	91	93	147	165	75
C2L (n = 9)	57	63	74	46	93	27
C2S (n = 18)	113	127	141	99	153	87
New (n = 42)	247	313	156	404	129	431
Reinstated condition (*N* = 37)						
C1L (n = 36)	307	137	194	250	310	134
C1S (n = 18)	123	99	115	107	148	74
C2L (n = 9)	61	50	66	45	87	24
C2S (n = 18)	107	115	148	74	141	81
NewL (n = 21)	110	149	88	171	69	190
NewS (n = 21)	90	169	87	172	78	181

**Table 17:** Dual-recollection multinomial model parameter estimates (standard errors) for aggregated data in Experiment 1

	C1L (n = 36)	C2L (n = 9)	C1S (n = 18)	C2S (n = 18)
Neutral condition				
*RT*	0.303 (0.035)	0.312 (0.065)	0.226 (0.043)	0.277 (0.047)
*RC*	0.208 (0.040)	0.228 (0.059)	0.123 (0.056)	0.210 (0.043)
*F*	0.185 (0.084)	0.450 (0.114)	0.401 (0.080)	0.176 (0.099)
*b*_*C1*_	0.441 (0.021)			
*b*_*C2*_	0.279 (0.019)			
*b*_*C1or2*_	0.230 (0.018)			
Reinstated condition				
*RT*	0.322 (0.041)	0.320 (0.064)	0.295 (0.043)	0.372 (0.047)
*RC*	0.209 (0.042)	0.097 (0.067)	0.030 (0.056)	0.184 (0.050)
*F*	0.233 (0.092)	0.520 (0.106)	0.304 (0.094)	0.000 (0.140)
*b*_*C1*_	0.425 (0.031)		0.347 (0.030)	
*b*_*C2*_	0.340 (0.029)		0.338 (0.029)	
*b*_*C1or2*_	0.266 (0.027)		0.299 (0.028)	

The italicized symbols are the parameters of the dual-recollection multinomial model: *RT* = target recollection, *RC* = context recollection, *F* = familiarity, *b* = response bias for Colour1?, Colour2?, and Color1 or Colour2? probe questions

In the neutral condition, the only significant difference was found between the guessing parameters, in particular, guessing “yes” was significantly higher when the participants were asked about Colour 1 than when they were asked about Colour 2, Δ*G*^2^(1) = 32.31, *p* < 0.001.

In the reinstated condition, the familiarity parameter (*F*) was higher for the configuration Colour1 and small font than for the equally frequent configuration Colour 2 and small font, Δ*G*^2^(1) = 4.45, *p* = 0.03. Moreover, the *b* guessing parameter was significantly higher for the distractors presented in large font when participants were asked about the more frequent Colour 1 than the less frequent Colour 2, Δ*G*^2^(1) = 3.96, *p* < 0.05. This result supports the conclusion that the response bias is affected by the base-rates. The between-group comparisons of memory parameters revealed no significant difference.

## Appendix 3: Results of descriptive measures used in Experiment 2

See Tables 18 and 19.

**Table 18:** Mean acceptance probabilities depending on the probe type in Experiment 2

Experimental conditions	Ci?	Cf?	Ciorf?
	*M* (*SD*)	*M* (*SD*)	*M* (*SD*)
Font colour – neutral			
Frequent (n = 30)	0.246 (0.137)	**0.832 (0.172)**	**0.846 (0.206)**
Infrequent (n = 18)	**0.809 (0.174)**	0.315 (0.205)	**0.839 (0.195)**
New (n = 18)	0.083 (0.124)		0.077 (0.147)
Colour name – neutral			
Frequent (n = 30)	0.190 (0.132)	**0.779 (.193)**	**0.828 (0.171)**
Infrequent (n = 18)	**0.770 (0.246)**	0.218 (0.184)	**0.833 (0.161)**
New (n = 18)	0.095 (0.168)		0.052 (0.090)
Font colour – reinstated			
Frequent (n = 30)	0.248 (0.228)	**0.730 (0.192)**	**0.833 (0.171)**
Infrequent (n = 18)	**0.809 (0.225)**	0.278 (0.249)	**0.827 (0.204)**
New (n = 18)	0.123 (0.199)	0.111 (0.196)	0.142 (0.280)
Colour name – reinstated			
Frequent (n = 30)	0.225 (0.165)	**0.736 (0.195)**	**0.800 (0.170)**
Infrequent (n = 18)	**0.696 (0.253)**	0.304 (0.261)	**0.804 (0.182)**
New (n = 18)	0.089 (0.160)	0.077 (0.124)	0.119 (0.212)

Results printed in boldface indicate correct responses. Ci? denotes probe question referring to an infrequently presented colour, Cf? denotes probe question referring to a frequently presented colour, Ciorf? denotes probe question about any colour (infrequent or frequent)

**Table 19:** Mean corrected acceptance probabilities for colour recognition in Experiment 2

	Ci?	Cf?	Ciorf?
	*M* (*SD*)	*M* (*SD*)	*M* (*SD*)
Font colour – neutral			
Frequent (n = 30)	0.163 (0.115)	**0.749 (0.259)**	**0.769 (0.261)**
Infrequent (n = 18)	**0.726 (0.234)**	0.232 (0.178)	**0.762 (0.254)**
Colour name – neutral			
Frequent (n = 30)	0.095 (0.122)	**0.684 (0.285)**	**0.776 (0.207)**
Infrequent (n = 18)	**0.675 (0.313)**	0.124 (0.137)	**0.782 (0.209)**
Font colour – reinstated			
Frequent (n = 30)	0.125 (0.206)	**0.619 (0.324)**	**0.691 (0.361)**
Infrequent (n = 18)	**0.685 (0.341)**	0.167 (0.217)	**0.685 (0.379)**
Colour name – reinstated			
Frequent (n = 30)	0.136 (0.163)	**0.658 (0.250)**	**0.681 (0.298)**
Infrequent (n = 18)	**0.607 (0.328)**	0.226 (0.245)	**0.684 (0.266)**

Results printed in boldface indicate correct responses

## Appendix 4: Results of multinomial modelling analyses using maximum-likelihood methods for Experiment 2

In Experiment 2, the sensitivity power analyses ensured a high test power (1 – β = 0.95) for the parameter comparisons across the conditions. With α = 0.05 and the number of responses across participants ranging from 1782 in the font colour reinstated condition to 1914 in the colour name neutral condition, small effect sizes ranging from *w* = 0.082 to 0.085 were detectable (G*Power 3; Faul et al. 2007). Parameter estimates were based on the aggregated data presented in Table 20.

**Table 20:** Aggregated frequencies of the responses in Experiment 2

	Ci?		Cf?		Ciorf?	
	Yes	No	Yes	No	Yes	No
Font colour – neutral condition (*N* = 28)						
Frequent (n = 30)	69	211	233	47	237	43
Infrequent (n = 18)	136	32	53	115	141	27
New (n = 18)	28	308			13	155
Colour name – neutral condition (*N* = 29)						
Frequent (n = 30)	55	235	226	64	240	50
Infrequent (n = 18)	134	40	38	136	145	29
New (n = 18)	33	315			9	165
Font colour – reinstated condition (*N* = 27)						
Frequent (n = 30)	67	203	197	73	225	45
Infrequent (n = 18)	131	31	45	117	134	28
New (n = 18)	18	144	20	142	23	139
Colour name – reinstated condition (*N* = 28)						
Frequent (n = 30)	63	217	206	74	224	56
Infrequent (n = 18)	117	51	51	117	135	33
New (n = 18)	13	155	15	153	20	148

The parameter estimates of the model are presented in Table 21. When the context recollection parameter was compared between the frequent versus the infrequent configurations, only one difference on a trend level was found—in the colour name reinstated condition, the context recollection parameter was higher for the frequent than the infrequent configuration Δ*G*^2^(1) = 3.82, *p* = 0.05. In the remaining three conditions, the differences were nonsignificant, Δ*G*^2^s(1) < 2.55. In the reinstated location conditions, the response bias parameter was not different depending on the probe question referring to the colour frequently versus infrequently paired with the reinstated location, Δ*G*^2^s(1) < 0.16.

**Table 21:** Dual-recollection multinomial model parameter estimates (standard errors) for aggregated data in Experiment 2

Parameter	Frequent configuration	Infrequent configuration	Frequent configuration	Infrequent configuration
	Font colour	Font colour	Colour name	Colour name
Neutral condition				
*RT*	0.558 (0.045)	0.589 (0.050)	0.406 (0.046)	0.433 (0.056)
*RC*	0.586 (0.034)	0.494 (0.047)	0.590 (0.033)	0.552 (0.045)
*F*	0.091 (0.177)	0.162 (0.200)	0.254 (0.128)	0.308 (0.153)
*b*_*C1*_ = *b*_*C2*_	0.083 (0.015)		0.095 (0.016)	
*b*_*C1or2*_	0.077 (0.021)		0.052 (0.017)	
Reinstated condition				
*RT*	0.409 (0.043)	0.537 (0.057)	0.410 (0.042)	0.455 (0.046)
*RC*	0.485 (0.039)	0.528 (0.048)	0.514 (0.037)	0.389 (0.051)
*F*	0.361 (0.111)	0.077 (0.221)	0.207 (0.127)	0.331 (0.133)
*b*_*Cf*_	0.111 (0.025)		0.077 (0.021)	
*b*_*Ci*_	0.123 (0.026)		0.089 (0.022)	
*b*_*Cf or i*_	0.142 (0.027)		0.119 (0.025)	

The italicized symbols are the parameters of the dual-recollection multinomial model: *RT* = target recollection, *RC* = context recollection, *F* = familiarity, and *b* = response bias depending on the probe question

## Appendix 5: Results of descriptive measures used in Experiment 3

See Tables 22 and 23.

**Table 22:** Mean acceptance probabilities depending on the probe type in Experiment 3

Experimental conditions	Ci?	Cf?	Ciorf?
	*M* (*SD*)	*M* (*SD*)	*M* (*SD*)
High disproportion			
Frequent (n = 48)	0.313 (0.167)	**0.785 (0.149)**	**0.795 (0.159)**
Infrequent (n = 12)	**0.664 (0.281)**	0.492 (0.280)	**0.836 (0.207)**
New (n = 30)	0.178 (0.211)	0.244 (0.278)	0.106 (0.162)
Low disproportion			
Frequent (n = 36)	0.372 (0.212)	**0.740 (0.202)**	**0.740 (0.202)**
Infrequent (n = 24)	**0.711 (0.194)**	0.355 (0.242)	**0.723 (0.241)**
New (n = 30)	0.228 (0.189)	0.209 (0.178)	0.172 (0.175)

Results printed in boldface indicate correct responses

**Table 23:** Mean corrected acceptance probabilities for colour recognition in Experiment 3

	Ci?	Cf?	Ciorf?
	*M* (*SD*)	*M* (*SD*)	*M* (*SD*)
High disproportion			
Frequent (n = 48)	0.134 (0.211)	**0.541 (0.315)**	**0.689 (0.227)**
Infrequent (n = 12)	**0.486 (0.355)**	0.248 (0.377)	**0.730 (0.246)**
Low disproportion			
Frequent (n = 36)	0.144 (0.245)	**0.530 (0.276)**	**0.568 (0.235)**
Infrequent (n = 24)	**0.483 (0.278)**	0.146 (0.278)	**0.551 (0.272)**

Results printed in boldface indicate correct responses

## Appendix 6: Results of multinomial modelling analyses using maximum-likelihood methods for Experiment 3

In Experiment 3, the sensitivity power analyses ensured a high test power (1 – β = 0.95) for the parameter comparisons. With the total number of 2880 responses across participants in the high or the low disproportion condition, small effect size (*w*) of 0.067 was detectable (Faul et al. 2007). Parameter estimates were based on the aggregated data presented in Table 24.

**Table 24:** Aggregated frequencies of the responses in Experiment 3

	Ci?		Cf?		Ciorf?	
	Yes	No	Yes	No	Yes	No
High disproportion condition (*N* = 32)						
Frequent (n = 48)	160	352	402	110	407	105
Infrequent (n = 12)	85	43	63	65	107	21
New (n = 30)	57	263	78	242	34	286
Low disproportion condition (*N* = 32)						
Frequent (n = 36)	143	241	284	100	284	100
Infrequent (n = 24)	182	74	91	165	185	71
New (n = 30)	73	247	67	253	55	265

The parameter estimates of the model are presented in Table 25. When the context recollection parameter was compared between the frequent versus the infrequent configurations, a significant difference was found in the high disproportion condition, Δ*G*^2^(1) = 14.501, *p* < 0.001, but not in the low disproportion condition, Δ*G*^2^(1) = 0.186. Moreover, in the high disproportion condition guessing parameter was significantly higher for the probe question about frequent than infrequent configuration, Δ*G*^2^(1) = 4.153, *p* = 0.04. When the context recollection parameter was compared between the high versus the low disproportion conditions, a significant difference was found only for the infrequent configuration of features, Δ*G*^2^(1) = 4.127, *p* = 0.04.

**Table 25:** Dual-recollection multinomial model parameter estimates (standard errors) for aggregated data in Experiment 3

Parameter	Frequent configuration	Infrequent configuration	Frequent configuration	Infrequent configuration
	High disproportion	High disproportion	Low disproportion	Low disproportion
*RT*	0.480 (0.034)	0.490 (0.049)	0.474 (0.036)	0.425 (0.042)
*RC*	0.454 (0.030)	0.199 (0.060)	0.373 (0.035)	0.348 (0.043)
*F*	0.192 (0.102)	0.551 (0.106)	0.045 (0.122)	0.474 (0.036)
*b*_*Cf*_	0.244 (0.024)		0.209 (0.023)	
*b*_*Ci*_	0.178 (0.021)		0.228 (0.023)	
*b*_*Cf or i*_	0.106 (0.017)		0.172 (0.021)	

The italicized symbols are the parameters of the dual-recollection multinomial model: *RT* = target recollection, *RC* = context recollection, *F* = familiarity, and *b* = response bias depending on the probe question

## Footnotes

1 We would like to thank Prof. Daniel W. Heck for pointing this problem out.

2 We would like to thank one of the reviewers for suggesting conducting such an experiment.

3 Taking into account 4 buffer items presented only at study, these differences in base-rates are a bit higher; 12:54 in the high disproportion condition and 24:40 in the low disproportion condition.

4 We would like to thank one of the reviewers for pointing this interesting area of investigation.
